# GRAF1a is a brain-specific protein that promotes lipid droplet clustering and growth, and is enriched at lipid droplet junctions

**DOI:** 10.1242/jcs.147694

**Published:** 2014-11-01

**Authors:** Safa Lucken-Ardjomande Häsler, Yvonne Vallis, Helen E. Jolin, Andrew N. McKenzie, Harvey T. McMahon

**Affiliations:** MRC Laboratory of Molecular Biology, Francis Crick Avenue, Cambridge CB2 0QH, UK

**Keywords:** GRAF1, ARHGAP26, Lipid droplet, BAR protein

## Abstract

Lipid droplets are found in all cell types. Normally present at low levels in the brain, they accumulate in tumours and are associated with neurodegenerative diseases. However, little is known about the mechanisms controlling their homeostasis in the brain. We found that GRAF1a, the longest GRAF1 isoform (GRAF1 is also known as ARHGAP26), was enriched in the brains of neonates. Endogenous GRAF1a was found on lipid droplets in oleic-acid-fed primary glial cells. Exclusive localization required a GRAF1a-specific hydrophobic segment and two membrane-binding regions, a BAR and a PH domain. Overexpression of GRAF1a promoted lipid droplet clustering, inhibited droplet mobility and severely perturbed lipolysis following the chase of cells overloaded with fatty acids. Under these conditions, GRAF1a concentrated at the interface between lipid droplets. Although GRAF1-knockout mice did not show any gross abnormal phenotype, the total lipid droplet volume that accumulated in GRAF1^−/−^ primary glia upon incubation with fatty acids was reduced compared to GRAF1^+/+^ cells. These results provide additional insights into the mechanisms contributing to lipid droplet growth in non-adipocyte cells, and suggest that proteins with membrane sculpting BAR domains play a role in droplet homeostasis.

## INTRODUCTION

Lipid droplets (LDs) are cytoplasmic lipid storage organelles. They are made of a neutral lipid core, mainly triglycerides and esterified cholesterol, surrounded by a monolayer of polar lipids (Fujimoto and Parton, 2011; Suzuki et al., 2011; Welte, 2007). Even though the brain has a low amount of LDs under resting conditions (Etschmaier et al., 2011; Zoula et al., 2003), LD content rises in brain tumours (Barba et al., 1999; Opstad et al., 2007; Rémy et al., 1997). LDs are also reported to accumulate in the brains of Alzheimer's patients (Gómez-Ramos and Asuncion Moran, 2007) and are proposed to be the site where aggregation of α-synuclein occurs in Parkinson's patients (Cole et al., 2002). The importance of LDs in the brain is also highlighted by the observation that seipin and spartin, proteins whose mutation is associated with neuronal diseases, target to LDs (Eastman et al., 2009; Szymanski et al., 2007). Deregulated lipid metabolism is also thought to be involved in other central nervous system disorders including Niemann–Pick diseases, schizophrenia and epilepsy (Adibhatla and Hatcher, 2007). Therefore, understanding the mechanisms controlling LD homeostasis in the brain is essential to determine the role of LDs in the onset and progression of a variety of neurological conditions.

Although details are currently lacking, LD formation is thought to start with the accumulation of neutral lipids between the two leaflets of the endoplasmic reticulum (ER) bilayer and the recruitment of LD-associated proteins (Brasaemle and Wolins, 2012; Fujimoto et al., 2008; Sturley and Hussain, 2012; Thiele and Spandl, 2008). Budding and scission would lead to small LDs surrounded by a lipid monolayer that could then travel on microtubules (Pol et al., 2004; Targett-Adams et al., 2003). LD mobility can contribute to lipid homeostasis by changing their cytoplasmic distribution (Orlicky et al., 2013), and by facilitating contact and lipid exchange with other LDs (Boström et al., 2005; Gong et al., 2011), and with the ER (Martin and Parton, 2008), mitochondria (Nan et al., 2006) and peroxisomes (Binns et al., 2006).

LD homeostasis has principally been studied in adipocytes, which, under resting conditions, have one large LD with a diameter of up to 100 µm. In contrast, non-adipocyte cells generally contain several LDs, with diameters in the low micrometre range (Suzuki et al., 2011). This not only results in a major difference in surface-to-volume ratio, and hence the accessibility to the LD core, but also a difference in the intrinsic curvature of LDs. Distinct mechanisms are thus likely to control LD homeostasis in adipocytes and non-adipocyte cells.

Proteins of the PAT family (perilipin, ADRP, TIP47, S3-12 and LSDP5, recently renamed PLIN1–PLIN5, respectively) share sequence similarity and the capacity to bind LDs (Bickel et al., 2009). Of these, only PLIN2 (ADRP) and PLIN3 (TIP47) are ubiquitously expressed. Both are thought to promote LD growth (Buers et al., 2009; Bulankina et al., 2009; Larigauderie et al., 2006; McIntosh et al., 2010; Sztalryd et al., 2006), but to label somewhat distinct LD populations (Ohsaki et al., 2006).

Proteins of the GTPase regulator associated with FAK (GRAF) family [GRAF1 (also called ARHGAP26), GRAF2 (also called ARHGAP10), GRAF3 (also called ARHGAP42) and oligophrenin-1 (OPHN1)] have N-terminal membrane-binding BAR and PH domains and a central GAP domain, with *in vitro* specificity for small Rho GTPases (Bai et al., 2013; Billuart et al., 1998; Hildebrand et al., 1996; Shibata et al., 2001; Taylor et al., 1999). GRAF1, GRAF2 and GRAF3 possess a C-terminal SH3 domain, whereas OPHN1 has a proline-rich domain instead. Relatively little is known about their functions. However, they are associated with several diseases, suggesting they are fulfilling important roles *in vivo*. In the case of GRAF1, reduced expression is reported in metastatic brain tumours from primary lung adenocarcinoma (Zohrabian et al., 2007), in multiple cases of myelodysplastic syndrome/acute myeloid leukemia (Bojesen et al., 2006; Borkhardt et al., 2000; Qian et al., 2011; Wilda et al., 2005), and in patients with X-linked alpha-thalassemia mental retardation syndrome (Barresi et al., 2010). In addition, anti-GRAF1 autoantibodies are found in patients with inflammatory cerebellar ataxia (Jarius et al., 2013; Jarius et al., 2010).

Two GRAF1 isoforms, GRAF1a and GRAF1b, are listed in databases, and a third isoform, GRAF1c, is reported (Barresi et al., 2010). GRAF1b associates with dynamic tubules and vesicles (Lundmark et al., 2008), but the roles of GRAF1a and GRAF1c have not been examined so far. We report here that, whereas GRAF1c shows a similar distribution to GRAF1b, GRAF1a specifically associates with LDs and is a regulator of LD homeostasis.

## RESULTS

### GRAF1a is a LD-associated protein enriched in neonatal brain

**Fig. 1.**
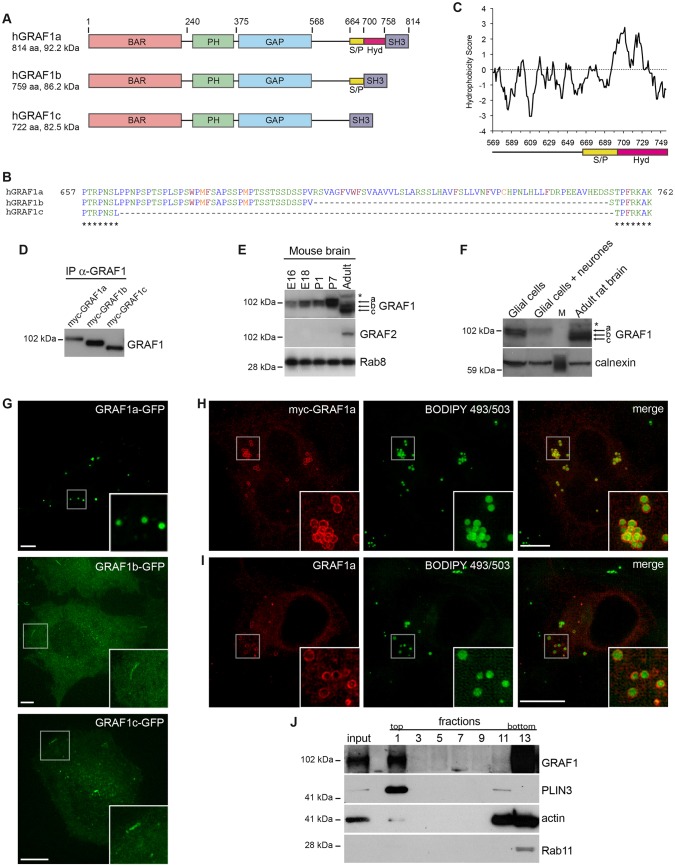
**GRAF1a is a LD-targeting isoform of GRAF1 expressed in neonates.** (A) Schematic representation of GRAF1 isoforms. S/P, serine- and proline-rich domain; Hyd, segment highly enriched in hydrophobic residues. (B) Sequence alignment of human (h)GRAF1a, GRAF1b and GRAF1c in the region of alternative splicing. Amino acids are coloured according to hydrophobicity (blue, hydrophobic and aliphatic; red, hydrophobic and aromatic; green, polar; orange, polar and sulfur-containing) (Protein Colourer, EMBL-EBI). (C) Kyte–Doolittle hydrophobicity analysis of amino acids 569–762 of hGRAF1a. Numbers refer to the beginning of the 9-amino-acid window used for the analysis (ProtScale interface, ExPASy, SIB). (D) Immunoprecipitation (IP) and western blot analysis of Myc-tagged GRAF1a, GRAF1b or GRAF1c, overexpressed in HeLa cells. (E) Western blot analysis of GRAF1 and GRAF2 in mouse brain extracts from E16 and E18 embryos, P1 and P7 neonates, and from an adult, showing the developmentally regulated expression of the GRAF proteins. Equal loading was verified on the same blot with an anti-Rab8 antibody. (F) Western blot analysis of GRAF1 in primary cultures of glial cells or of glial cells and neurones, and in adult rat brain. M, marker lane. Equal loading was checked on the same blot with an anti-calnexin antibody. Results in E and F are representative of more than two independent experiments. Bands corresponding to isoforms a, b and c of GRAF1 are indicated by arrows. * indicates a non-specific band absent from the immunoprecipitations (see supplementary material Fig. S1A,B). (G) Single focal plane images of live HeLa cells expressing GFP-tagged GRAF1a, b or c (see corresponding supplementary material Movies 1–3). Scale bars: 15 µm. (H,I) Maximum intensity projections of images acquired by structured illumination microscopy (SIM) of HeLa cells expressing Myc–GRAF1a (H) or untagged GRAF1a (I), respectively, stained with anti-Myc or anti-GRAF1 antibodies, and using BODIPY 493/503 to visualize LDs. Scale bars: 10 µm. Boxed areas in G–I are enlarged at the bottom right of each image. (J) Separation of cytoplasmic components of oleic-acid-fed primary glial cells by density gradient centrifugation. The input and odd fractions were analysed by western blotting, showing relative enrichment of endogenous GRAF1a and PLIN3 at the top of the gradient (fraction 1). Actin and Rab11 were used as negative controls.

Previous studies have shown that GRAF1 is enriched in mammalian brain (Hildebrand et al., 1996; Taylor et al., 1998). We therefore amplified *GRAF1* sequences from a brain cDNA library. We sequenced 20 clones and identified three splice variants, GRAF1a, GRAF1b and GRAF1c. The proteins encoded differed in a region with no predicted secondary structure preceding the terminal SH3 domain (Fig. 1A). The most abundant cDNAs corresponded to the shorter isoforms (11/20 clones for GRAF1b; 8/20 for GRAF1c). In comparison to GRAF1b, GRAF1c lacked a 37-amino-acid stretch enriched in serine and proline residues (S/P, Fig. 1B). In comparison to GRAF1b, GRAF1a contained an additional 55-amino-acid segment highly enriched in hydrophobic residues (Hyd, Fig. 1A–C). Only 1/20 clones corresponded to GRAF1a, suggesting that it is the least abundant in adult brain.

GRAF1a, GRAF1b and GRAF1c could be discriminated by their sizes (Fig. 1D). In agreement with the PCR data, in adult mouse or rat brain protein extracts, GRAF1 migrated as two major bands, corresponding to the molecular masses of GRAF1b and GRAF1c (Fig. 1E; supplementary material Fig. S1A,B). Interestingly, however, the pattern of GRAF1 expression was different in developing mouse brain. Expression of the longer isoform (GRAF1a) started before embryonic day 16 (E16) and increased postnatally, after which there was a switch to shorter isoforms in adults (Fig. 1E). Immunoprecipitation of postnatal day 7 (P7) brain extracts with an anti-GRAF1 antibody resulted in a protein with the same molecular mass as GRAF1a (supplementary material Fig. S1A). GRAF1b and GRAF1c are thus the major GRAF1 isoforms in adult brain, whereas GRAF1a is abundant in neonates. GRAF1a was also predominant in primary cells isolated from the brains of E18 embryos (Fig. 1F). At equal protein amounts, glial cultures devoid of neurones contained more GRAF1 than co-cultures, suggesting that GRAF1a is enriched in glial cells (Fig. 1F).

When overexpressed, GFP-tagged GRAF1b and GRAF1c labelled tubules and small vesicles (Fig. 1G). As previously reported for GRAF1b (Lundmark et al., 2008), they were dynamic (data not shown). In striking contrast, GRAF1a mostly labelled the membrane surrounding spherical cytoplasmic inclusions (Fig. 1G), which were relatively immobile (data not shown). Similar structures were labelled when GRAF1a–GFP was transfected in rat pheochromocytoma PC12, human neuroblastoma SH-SY5Y, murine NIH 3T3 fibroblasts and African green monkey epithelial BSC1 cells (supplementary material Fig. S1C). Using Myc-tagged GRAF1a and the neutral lipid probe BODIPY 493/503 (Fig. 1H), these organelles were identified as LDs. Similarly, overexpressed untagged GRAF1a was found on LDs in HeLa cells (Fig. 1I), human glioblastoma U-87 MG cells (supplementary material Fig. S1D) and primary glial cells (supplementary material Fig. S1E,F). A reticular background staining, typical of the ER, was seen upon staining with anti-GRAF1 antibodies when the LD number was low. In order to examine the subcellular distribution of endogenous GRAF1a, cytoplasmic extracts of primary glial cells were fractionated on a sucrose gradient. Similar to what has been previously reported (Brasaemle and Wolins, 2006), LDs were at the top of the gradient, as fraction 1 was enriched in PLIN3 (Fig. 1J). GRAF1a was also enriched in fraction 1 (Fig. 1J). Collectively, these experiments show that GRAF1a is a GRAF1 isoform enriched in the brain of neonates that associates with LDs under resting conditions, in a variety of cell lines and primary cells.

### Specific association of GRAF1a with LDs relies both on an internal hydrophobic linker and its BAR and PH domains

**Fig. 2.**
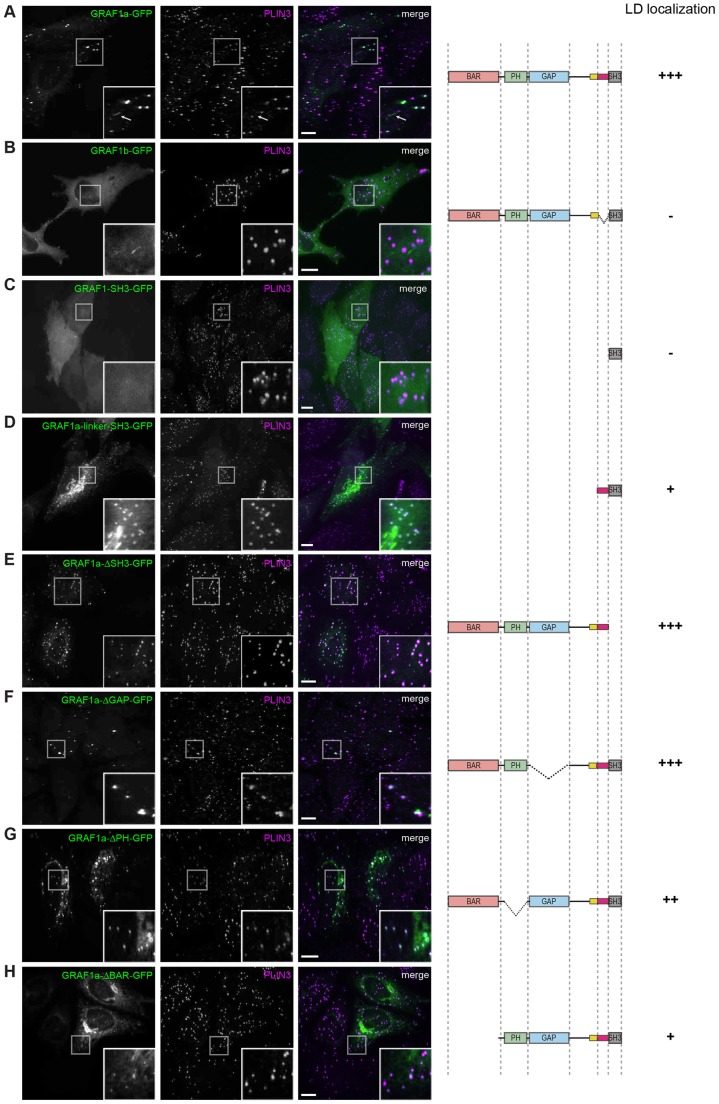
**The hydrophobic segment of GRAF1a, its BAR and its PH domains are required for specific targeting to LDs.** (A–H) HeLa cells, transfected with the indicated GFP-tagged GRAF1 constructs, were incubated with oleic acid for 1 h and processed for immunofluorescence with an anti-PLIN3 antibody to examine their LD localization. Confocal stacks are shown. A boxed area is enlarged at the bottom right of each image. In A, an arrow points to a GRAF1a tubule. Scale bars: 15 µm.

Microscopy observation showed that GRAF1a and GRAF1b are both membrane-binding proteins, but they do not target the same intracellular compartments. In addition, unlike GRAF1b, the association of GRAF1a with membranes was resistant to sodium carbonate extraction (supplementary material Fig. S1G). In comparison to GRAF1b, GRAF1a has a short additional internal linker (Fig. 1A). This linker therefore mediates binding to membranes through hydrophobic interactions. In addition, given that, unlike GRAF1a (Fig. 2A), GRAF1b did not associate with LDs (Fig. 2B), this linker is required for LD targeting. It was also sufficient to drive the SH3 domain of GRAF1 to LDs (Fig. 2C,D), although, in comparison to full-length GRAF1a, the targeting was less specific.

Deletion of the SH3 or of the GAP domain of GRAF1a did not perturb its distribution (Fig. 2E,F). However, deletion of either membrane-binding region led to background binding to networks of internal membranes (Fig. 2G,H). GRAF1a-ΔPH showed extensive colocalization with the mitochondrial protein COX IV (supplementary material Fig. S1H). GRAF1a-ΔBAR colocalized with the Golgi protein GM130, but also with COX IV and the ER protein calreticulin (supplementary material Fig. S1I). When LDs were enlarged by incubating cells with oleic acid, GRAF1a-ΔPH acquired a more exclusive LD localization (supplementary material Fig. S1J). GRAF1a-ΔBAR, however, still showed residual binding to other membranes, suggesting that it had a substantially reduced affinity for LDs (supplementary material Fig. S1K).

Western blot analysis of cell extracts confirmed that there was little cleavage of the overexpressed proteins and that the GFP fusions examined thus corresponded to the GRAF1a deletion mutants (supplementary material Fig. S1L). In addition, fractionation of cytoplasmic extracts of transfected U-87 MG cells showed that GRAF1a–GFP, GRAF1a-ΔPH–GFP, GRAF1a-linker-SH3–GFP and GRAF1a-ΔBAR–GFP were all found in the LD-enriched fraction, although the latter two were present to a lesser extent (supplementary material Fig. S1M).

These experiments show that no single domain of GRAF1a is sufficient for specific LD binding. Its hydrophobic linker is the only necessary region, but its BAR and PH domains are both required to restrict the distribution of GRAF1a to the LD surface.

### GRAF1a overexpression induces an increase in LD volume

**Fig. 3.**
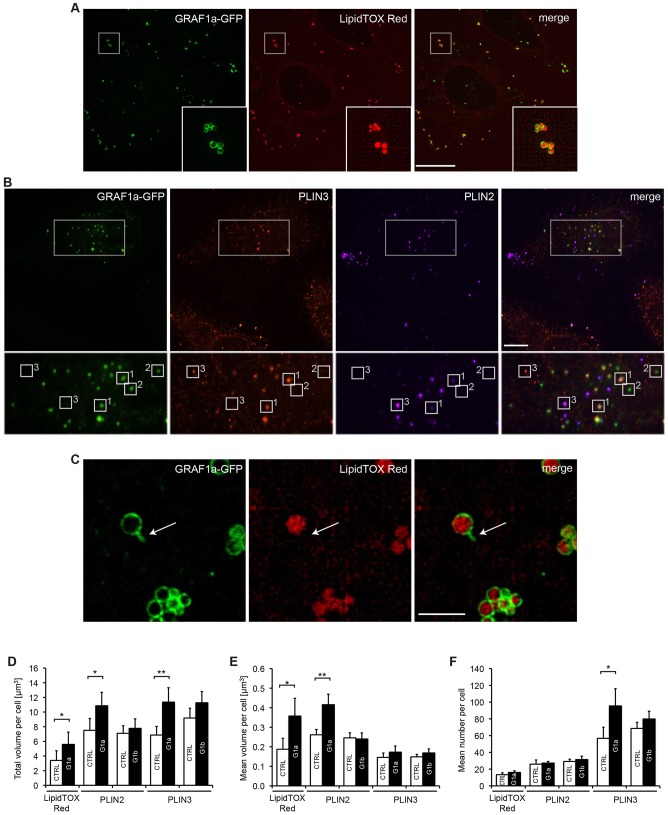
**Overexpression of GRAF1a leads to an increase in LD volume.** (A–C) HeLa cells transfected with GRAF1a–GFP were either stained with LipidTOX Red (A,C) or processed for immunofluorescence analysis with anti-PLIN2 and anti-PLIN3 antibodies (B) to visualize the presence of GRAF1a in different sub-populations of LDs. A boxed area is enlarged at the bottom of each image. (A,C) Cells were visualized by SIM. (A) Maximum intensity projection. Scale bar: 15 µm. (C) Single focal plane. An arrow points at a GRAF1a tubule extending from the surface of the LD. Scale bar: 2 µm. In B, images are of confocal stacks. Boxes numbered 1 indicate LDs containing GRAF1a, PLIN2 and PLIN3, boxes numbered 2, LDs containing only GRAF1a, and boxes numbered 3, LDs devoid of GRAF1a. Scale bar: 15 µm. (D–F) HeLa cells transfected with GRAF1a–GFP (G1a) or GRAF1b–GFP (G1b) were fixed after 48 h. As indicated, LDs were identified after staining with LipidTOX Red, or immunofluorescence analysis with anti-PLIN2 or anti-PLIN3 antibodies. For each condition, the total LD volume (D), the mean LD volume (E) and the mean LD number (F) per transfected and untransfected (CTRL) cell was quantified. LipidTOX Red, 156 CTRL and 233 G1a in 5 independent experiments; PLIN2, 181 CTRL and 243 G1a in 13 independent experiments, 193 CTRL and 111 G1b in 7 independent experiments; PLIN3, 152 CTRL and 204 G1a in 11 independent experiments, 213 CTRL and 189 G1b in 8 independent experiments. Results are mean±s.e.m. *P<0.05; **P<0.01.

Within a cell, the LD population is heterogeneous in terms of lipid and protein composition; this has been proposed to reflect the fact that individual LDs can be in different metabolic states (Martin et al., 2005; Ohsaki et al., 2006; Storey et al., 2011; Wolins et al., 2005). Under resting conditions, when HeLa cells were transfected with GRAF1a, the protein was found on most LDs, as visualized with lipophilic probes, such as BODIPY 493/503 (Fig. 1H,I; supplementary material Fig. S1D–F) or LipidTOX Red (Fig. 3A). If LDs were detected with anti-PLIN2 and anti-PLIN3 antibodies, some LDs had the three proteins (Fig. 3B, boxes labelled 1), some were only positive for GRAF1a (Fig. 3B, boxes labelled 2), and a few were devoid of GRAF1a (Fig. 3B, boxes labelled 3). As previously reported, some LDs contained PLIN2 and not PLIN3, and vice-versa (Fig. 3B). PLIN3 was slightly more abundant on GRAF1a-positive LDs than PLIN2 (supplementary material Fig. S2A), but neither protein was found on all GRAF1a LDs. These observations thus extend LD heterogeneity to the presence or absence of GRAF1a.

In some cells, in addition to LDs, GRAF1a was found on small tubules that sometimes seemed to extend from LDs (Fig. 2A; Fig. 3C). These tubules, however, were not stained with neutral lipid probes (Fig. 3C; supplementary material Fig. S2B), did not contain PLIN2 (supplementary material Fig. S2C) or PLIN3 (Fig. 2A), and were not induced during phases of LD growth (see below). Whether these tubules were artefacts of the overexpression of GRAF1a leading to its association with endogenous GRAF tubules or a specific structure formed by GRAF1a will require further exploration.

When the size and number of LDs, identified by staining with LipidTOX Red, or as PLIN2-positive or PLIN3-positive objects, were quantified and compared between untransfected and GRAF1a–GFP-expressing cells, GRAF1a induced a significant increase in total LD volume (Fig. 3D). In the case of LipidTOX Red and PLIN2, it did so through an increase in the mean volume of LDs (Fig. 3E), whereas it mostly increased the number of PLIN3-positive structures (Fig. 3F). Similar results were obtained when LipidTOX Red, BODIPY 493/503 or PLIN3-positive LD properties were compared between Myc–GRAF1a-transfected and untransfected cells (supplementary material Fig. S2D–F), ruling out an effect of the tag on this phenotype. In addition, as GRAF1a overexpression did not change the efficiency of binding of PLIN2 and PLIN3 to LDs (supplementary material Fig. S2G), nor did overexpressed GRAF1a sterically hinder access to the LD surface. This size increase was specific to the LD-targeting isoform of GRAF1, and was not caused by transfection per se, because GRAF1b expression did not change LD properties (Fig. 3D–F; supplementary material Fig. S2H).

### GRAF1a overexpression promotes an increase in LD size upon fatty acid loading of cells

**Fig. 4.**
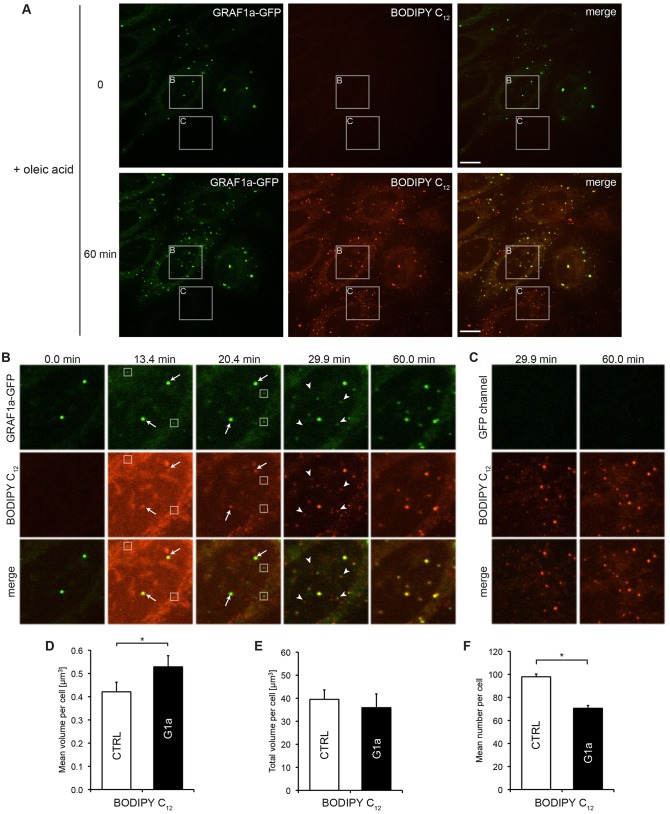
**GRAF1a induces an increase in mean LD volume upon fatty acid loading of cells.** HeLa cells transfected with GRAF1a–GFP were incubated with oleic acid and BODIPY C_12_. (A–C) Time-lapse movies of live cells were acquired on a single focal plane. (A) Still images at 0 and 60 min. Scale bars: 15 µm. (B,C) Still images of regions boxed in A at the indicated time-points showing transfected (B) and untransfected (C) cells. In B, arrows point to pre-existing LDs, boxes are placed around GRAF1a-positive nascent LDs, and arrowheads indicate BODIPY C_12_-positive nascent LDs. (D–F) Cells were fixed after 1 hour. LDs were identified as BODIPY-C_12_-positive droplets. The mean LD volume (D), total LD volume (E) and mean LD number (F) per transfected (G1a) and untransfected (CTRL) cell were quantified in three independent experiments using 85 CTRL and 106 G1a cells. Results are mean±s.e.m. *P<0.05.

In order to follow the behaviour of GRAF1a during LD formation, GRAF1a–GFP-transfected cells were incubated with oleic acid and BODIPY 558/568 C_12_ (hereafter called BODIPY C_12_), a fluorescent fatty acid analogue that incorporates into LDs with similar kinetics to unmodified fatty acids (Liao et al., 2005; Wang et al., 2010) (Fig. 4A,B; supplementary material Movie 1). Within 5–15 min of addition, pre-existing LDs were labelled with BODIPY C_12_ (Fig. 4B, arrows). At this time, new GRAF1a-positive puncta were seen (Fig. 4B, boxed). After 20–30 min, small BODIPY-C_12_-positive LDs appeared, sometimes co-stained with GRAF1a, and sometimes not (Fig. 4B, arrowheads). Thereafter, this large number of small LDs transformed into fewer larger LDs, which, after 1 h, were almost all positive for GRAF1a. Interestingly, in untransfected cells, the density of LDs did not diminish as clearly, suggesting that this decrease was stimulated by GRAF1a (Fig. 4C; supplementary material Fig. S2I).

When the size and number of LDs was quantified in fixed cells after a 1-h incubation with oleic acid, the mean LD volume was increased in cells overexpressing GRAF1a (Fig. 4D; supplementary material Fig. S2J). However, this did not lead to an increase in total LD volume (Fig. 4E) as the number of distinct LDs was reduced (Fig. 4F). Similarly to resting cells, the efficiency of binding of PLIN3 to LDs was not altered by GRAF1a (supplementary material Fig. S2K). Overexpression of GRAF1a therefore does not change the capacity of cells to take up fatty acids, or the efficiency of incorporation into LDs, but rapidly leads to a smaller number of larger LDs.

### Overexpression of GRAF1a reduces LD motility

**Fig. 5.**
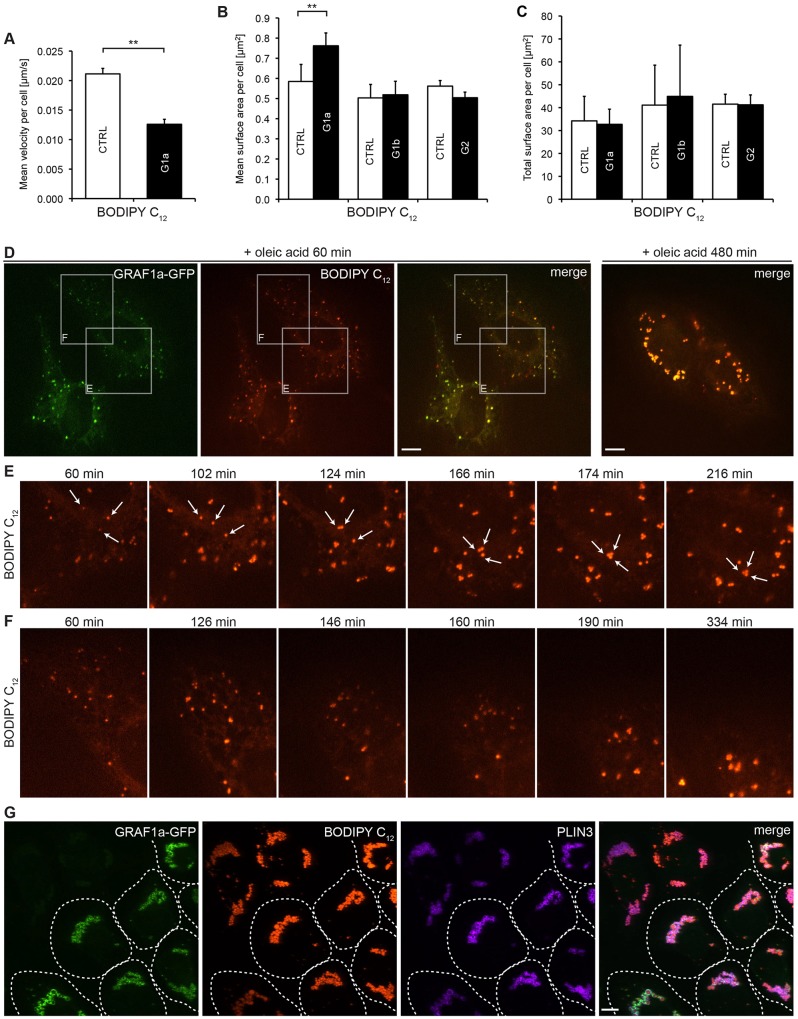
**Overexpression of GRAF1a slows down LDs and potentiates the clustering induced by fatty acid loading.** HeLa cells transfected with GFP-tagged GRAF1a (G1a), GRAF1b (G1b) or GRAF2 (G2) were incubated with oleic acid and BODIPY C_12_. (A–F) Time-lapse movies of live cells were acquired on a single focal plane. (A) Individual BODIPY-C_12_-positive LDs were identified and tracked. The mean LD velocity in untransfected (CTRL) and GRAF1a-transfected (G1a) cells was quantified in three independent experiments on a total of 53 CTRL and 27 G1a cells, 2–5 h after oleic acid addition. (B,C) Mean (B) and total (C) surface area of BODIPY-C_12_-positive LDs per untransfected (CTRL) and transfected cell quantified from 54 CTRL, 34 G1a; 16 CTRL, 27 G1b; and 45 CTRL, 50 G2 cells in four, two and two independent experiments, respectively. Results are mean±s.e.m. **P<0.01. (D–F) Still images of cells, starting 60 min after oleic acid addition. Boxed areas in D are enlarged in E and F showing LD clustering because of individual movements (E) or of large-scale cytoplasmic rearrangements (F). In E, arrows point to individual LDs followed during the course of the imaging. (G) Immunofluorescence analysis of cells fixed 24 h after oleic acid addition, showing clustering of LDs in the perinuclear region of both untransfected and GRAF1a–GFP-expressing cells. The latter are surrounded by dashed lines. Images are projections of confocal stacks. Scale bars: 15 µm (D,G).

The precise mechanism responsible for LD growth is not known, but it is thought to proceed through a combination of local lipid synthesis, lipid transfer and LD fusion (Yang et al., 2012). The latter two will depend on the ability of a LD to reach its target and tether itself onto it, and are thus tightly linked to LD mobility. Overexpression of GRAF1a induced a significant decrease in mean LD velocity (Fig. 5A; supplementary material Fig. S2L, Movie 2). As above with fixed cells, GRAF1a overexpression also induced an increase in mean LD area, but not of total LD area (Fig. 5B,C). This was specific to GRAF1a, because it was neither induced by GRAF1b nor by GRAF2 (Fig. 5B,C).

When cells were incubated with fatty acids, LDs were often seen as tight clusters (Fig. 5D). This was particularly striking in cells overexpressing GRAF1a (supplementary material Fig. S2M) and partially explained the increase in mean LD size. Indeed, LDs were so tightly bound to each other that they sometimes could not be discriminated as independent objects during the analysis of the images. LDs came together as a result of directed movements (Fig. 5E) or of large-scale cytoplasmic rearrangements (Fig. 5F), and remained bound to each other, often without clearly showing signs of fusion. As a result, when cells were incubated with fatty acids for 24 h and imaged, LDs were clustered in the perinuclear region. This was seen both in control and in GRAF1a-overexpressing cells (Fig. 5G; supplementary material Fig. S2N). At this stage, LDs did not disperse upon treatment with nocodazole, suggesting that it could not be explained by tethering to the microtubule network (supplementary material Fig. S2O).

These experiments thus show that during phases of LD growth, LDs cluster. GRAF1a overexpression limits LD mobility and potentiates their clustering.

### GRAF1-knockout mice do not show a strong abnormal phenotype

**Fig. 6.**
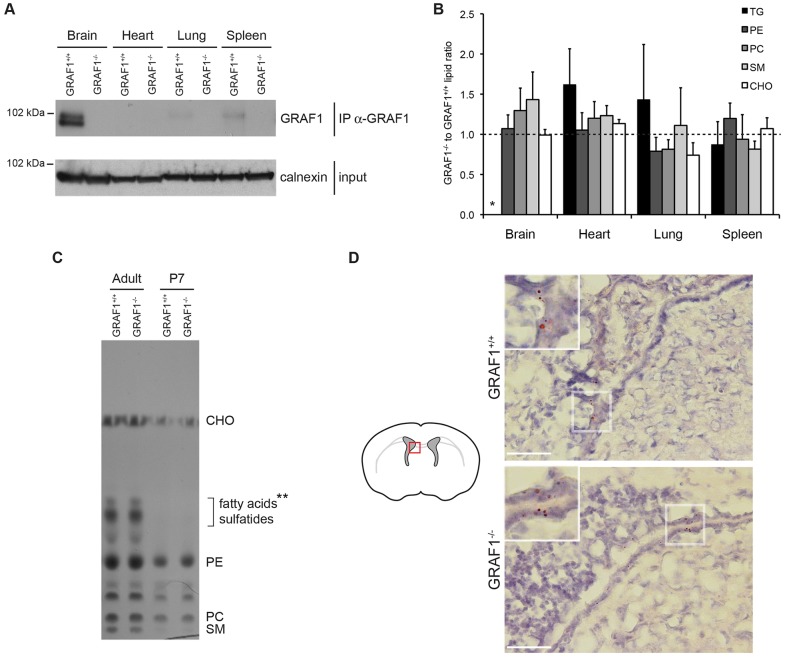
**GRAF1^−/−^ mice do not show a strong gross phenotype.** (A) Immunoprecipitation of GRAF1 in brain, heart, lung and spleen extracts of GRAF1^+/+^ and GRAF1^−/−^ mice, showing its enrichment in the brain, and its knockout in GRAF1^−/−^ mice. Equal protein amount in the input was checked by western blotting using an anti-calnexin antibody. The result shown is representative of more than three pairs of animals. (B,C) Adult brain, heart, lung and spleen (B), or adult and P7 brain (C) lipid extracts from GRAF1^+/+^ and GRAF1^−/−^ mice were analysed by TLC. (B) Means±s.e.m. of the spot intensity ratio of triglyceride (TG), phosphatidylethanolamine (PE), phosphatidylcholine (PC), sphingomyelin (SM) and cholesterol (CHO) between independent pairs of GRAF1^−/−^ and GRAF1^+/+^ littermates, showing no significant difference in their composition (*n* = 3) (see supplementary material Fig. S3D). As the triglyceride spot intensity of brain samples is at background level, the corresponding mean was not included (* in B). The spots indicated by ** in C co-migrate with fatty acids, but because they are absent from P7 brain and from all other organs (supplementary material Fig. S3D), they probably correspond to sulfatides, a major component of myelin (Cohen et al., 2005; Paglia et al., 2010). (D) Oil Red O staining of GRAF1^+/+^ and GRAF1^−/−^ brain sections from P7 pups. Boxed areas are magnified at the top left of each image. Scale bars: 50 µm.

In order to examine whether GRAF1a plays a role in LD growth *in vivo*, a knockout mouse was engineered (supplementary material Fig. S3A; Fig. 6A). Knockout pups were indistinguishable from their wild-type littermates. GRAF1-knockout mice developed normally, and were fertile and of similar weights as wild-type animals (supplementary material Fig. S3B). Histological analysis of various organs revealed no abnormality in tissue architecture (supplementary material Fig. S3C). GRAF1 is therefore not essential for mouse development. Thin-layer chromatography (TLC) analysis showed that overall, there were no significant lipid differences between GRAF1^+/+^ and GRAF1^−/−^ mice, whether looking at tissues from P7 neonates or adults (Fig. 6B,C; supplementary material Fig. S3D–F). In addition, triglyceride levels in their brains were low. In agreement with this, Oil Red O staining of P7 brain sections showed only a few LDs in cells lining the ventricles and no gross difference between knockout and wild-type animals (Fig. 6D).

Partial compensation of GRAF1b and GRAF1c by GRAF2, which has a similar domain organization and was also expressed in adult brain (Fig. 1E) could not be ruled out, but there was no change in its expression level (supplementary material Fig. S3G). GRAF2 was, however, unlikely to compensate for GRAF1a, not only because GRAF2 did not target LDs (supplementary material Fig. S3H), but also because it was absent from brain extracts of P7 pups, where GRAF1a was abundant (Fig. 1E).

### Primary GRAF1^−/−^ glial cells show impaired LD accumulation upon incubation with oleic acid

**Fig. 7.**
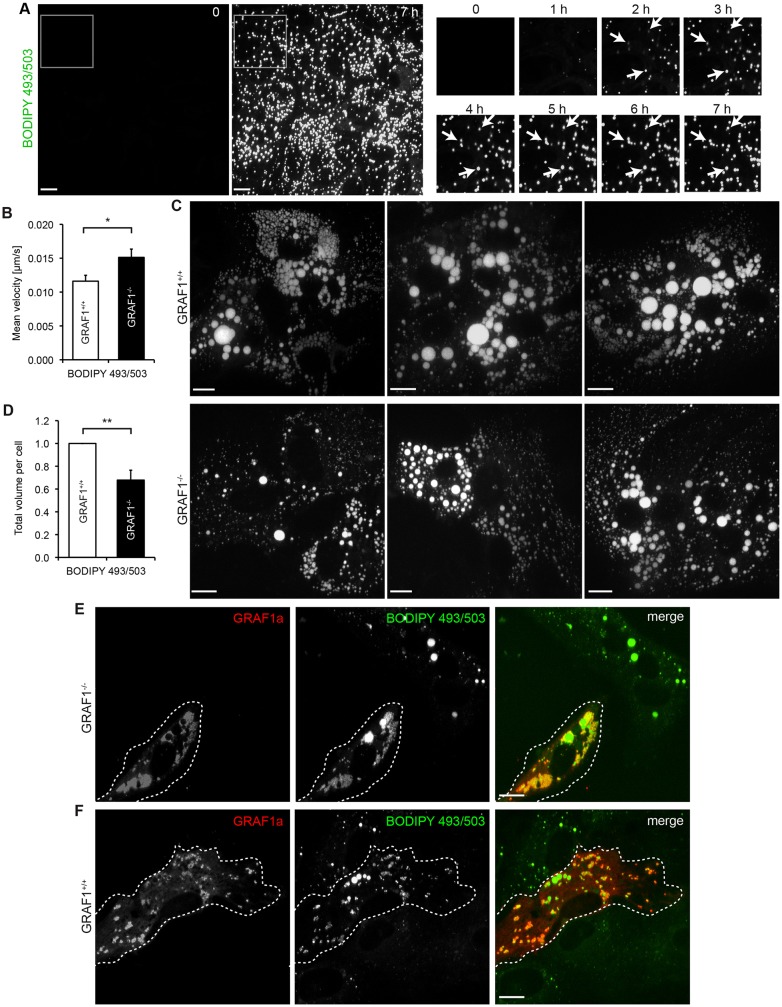
**GRAF1a contributes to LD growth in primary glial cells.** (A) Still images of live primary glial cells on a single focal plane, starting upon addition of oleic acid and BODIPY 493/503. The boxed area is enlarged on the right at 1 h intervals. Arrows are placed at fixed positions and point at three LDs remaining essentially immobile during the imaging. (B) Mean LD velocity per cell quantified in primary glial cells isolated from eight GRAF1^+/+^ (16 cells) and eight GRAF1^−/−^ (19 cells) embryos and stained with BODIPY 493/503, 10 h after oleic acid addition. Results are mean±s.e.m. *P<0.05 (unpaired Student's *t*-test). (C,D) Primary GRAF1^+/+^ and GRAF1^−/−^ glial cultures were incubated with oleic acid for 48 h, fixed and stained with BODIPY 493/503. (C) Representative images of cells isolated from three wild-type and three knockout embryos. (D) The total LD volume per cell was quantified for 840 GRAF1^−/−^ and 494 GRAF1^+/+^ primary glial cells isolated from 9 GRAF1^−/−^ and 9 GRAF1^+/+^ embryos in four independent litters. For each embryo, the mean total LD volume per cell was quantified. It was then averaged for all GRAF1^+/+^ and for all GRAF1^−/−^ embryos within the same litter and normalized by the value obtained for wild-type embryos. Results are mean±s.e.m. **P<0.01. (E,F) Primary GRAF1^−/−^ (E) and GRAF1^+/+^ (F) glial cells were transfected with untagged GRAF1a, incubated with oleic acid for 24 (F) or 48 (E) h and processed for immunofluorescence with an anti-GRAF1 antibody and BODIPY 493/503, showing GRAF1a-induced LD clumping and accumulation. Transfected cells are surrounded by dashed lines. (C,E,F) Images correspond to projections of confocal stacks. Scale bars: 15 µm.

We decided to compare LD accumulation in primary glial cultures isolated from GRAF1^+/+^ and GRAF1^−/−^ embryos. In agreement with the histology analysis, GRAF1^+/+^ and GRAF1^−/−^ cells contained few LDs under resting conditions (supplementary material Fig. S3I). Upon incubation with oleic acid there was an accumulation of LDs in primary glia (Fig. 7A). When imaged during their growth phase, LDs were strikingly immobile (Fig. 7A) but knocking out GRAF1 increased the mean LD velocity (Fig. 7B). Live imaging showed that in GRAF1^−/−^ cells, LDs had more freedom of movement around their point of anchor (supplementary material Movie 3). The presence of GRAF1 thus restricts LD mobility.

When primary glia were incubated with oleic acid for 48 h, the total LD volume in GRAF1^−/−^ cells was significantly smaller than in GRAF1^+/+^ cells (Fig. 7C,D). Transfection of untagged GRAF1a in GRAF1^−/−^ cells promoted LD clustering and accumulation (Fig. 7E; supplementary material Fig. S3J). When transfected into GRAF1^+/+^ cells, GRAF1a also induced LD clumping 24 h after oleic acid addition (Fig. 7F), but did not further enhance the clustering seen at 48 h (supplementary material Fig. S3K). These experiments thus show that, similar to what we observed in HeLa cells, GRAF1a regulates LD mobility, facilitates inter-LD contacts and contributes to LD growth in primary glial cells.

### Overexpression of GRAF1a prevents LD dispersion after the washout of fatty acids

**Fig. 8.**
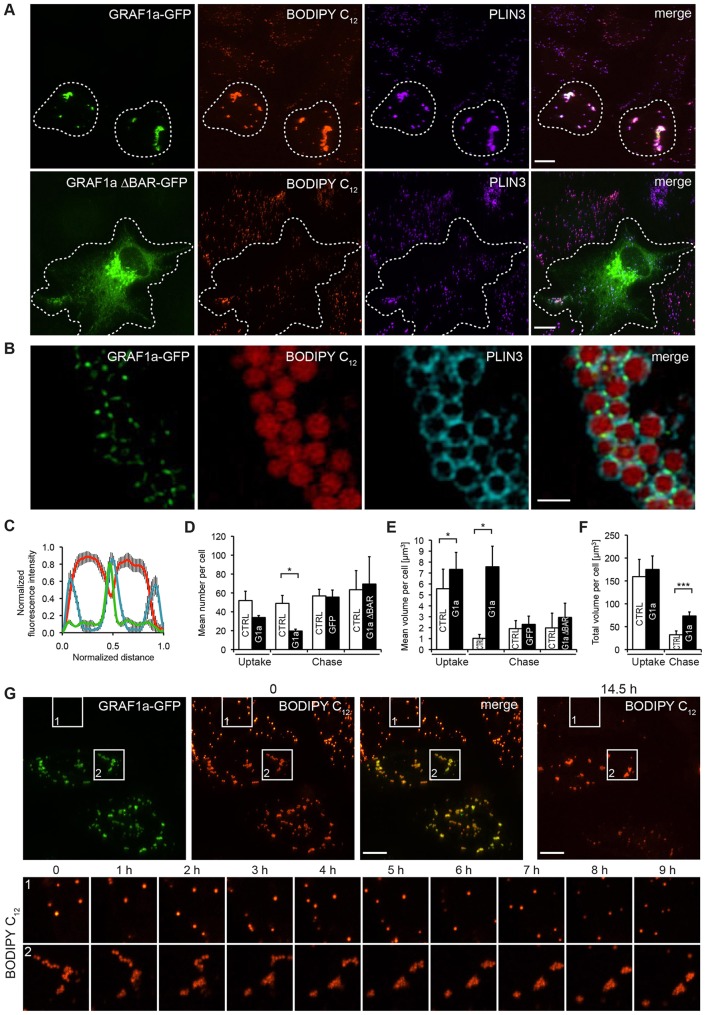
**Overexpression of GRAF1a prevents LD spreading and inhibits lipolysis during the washout of fatty acids from overloaded cells.** (A–F) HeLa cells, transfected with GFP, GRAF1a–GFP (G1a), or GRAF1a-ΔBAR–GFP (G1a ΔBAR) were incubated with oleic acid and BODIPY C_12_ for 24 h. Cells were then either directly fixed (Uptake), or fatty acids were first washed out for another 24 h (Chase). (A–C) After the chase, cells were analysed by immunofluorescence with an anti-PLIN3 antibody. (A) Projections of confocal stacks are shown. Transfected cells are surrounded by dashed lines. (B,C) Cells were imaged by SIM. (B) Single focal plane images of a GRAF1a–GFP-transfected cell, showing a discontinuous distribution of GRAF1a on the surface of LDs. Scale bar: 1 µm. (C) Analysis of BODIPY C_12_ (red), PLIN3 (blue), and GRAF1a–GFP (green) fluorescence intensity profiles on lines dissecting pairs of LDs (*n* = 10), showing the accumulation of GRAF1a at LD junctions. (D–F) Mean number (D), mean volume (E) and total volume (F) of BODIPY-C_12_-positive LD clusters per transfected and untransfected (CTRL) cell quantified in eight (G1a Uptake), five (G1a Chase), four (GFP) and three (G1a ∶BAR) independent experiments. Cell numbers: Uptake, 127 CTRL, 186 G1a; Chase, 132 CTRL, 160 G1a; 150 CTRL, 70 GFP; 154 CTRL, 58 G1a ΔBAR. Results are mean±s.e.m. *P<0.05; ***P<0.001. (G) Still images of live HeLa cells, transfected with GRAF1a–GFP and pre-incubated with oleic acid and BODIPY C_12_ for 8 h, starting upon fatty acid removal. (Top) 2D projections of confocal stacks at the beginning (0) and end (14.5 h) of the chase. (Bottom) Single focal plane images of the boxed areas at 1 h intervals, showing mobile independent LDs in untransfected cells (box 1) and clumped immobile LDs in GRAF1a-expressing cells (box 2). Scale bars: 15 µm.

After 24 h of oleic acid loading, LDs accumulated *en masse* in a pericentriolar region (Fig. 5G). When fatty acids were removed from the medium, LDs dispersed (Fig. 8A). This is similar to what happens in PLIN1-transfected fibroblasts when lipolysis is stimulated (Marcinkiewicz et al., 2006). Strikingly, after a 24-h wash, dispersion did not occur in cells overexpressing GRAF1a–GFP (Fig. 8A). This was also seen with untagged GRAF1a (supplementary material Fig. S4A) but not with GFP (supplementary material Fig. S4B). Imaging of cells by super-resolution structured illumination microscopy (SIM) showed that GRAF1a proteins were clustered at inter-LD contacts, whereas PLIN3 was distributed on the whole surface of the LDs, suggesting that GRAF1a participates in tethering LDs together (Fig. 8B,C). In comparison to untransfected cells, expression of GRAF1a led to the tight association of LDs into a smaller number of larger clusters, whether LDs were identified as BODIPY-C_12_-positive structures (Fig. 8D,E) or PLIN3-positive objects (supplementary material Fig. S4C,D), but did not alter the binding efficiency of PLIN3 to LDs (supplementary material Fig. S4F). In addition, GRAF1a overexpression attenuated the decrease in total LD volume seen upon fatty acid washout (Fig. 8F; supplementary material Fig. S4E), suggesting an inhibition of lipolysis. In agreement with this, although there was no difference in the amount of triglycerides stored by cells after the uptake, the triglyceride levels remaining in GRAF1a-overexpressing cells after the chase was higher than in controls (supplementary material Fig. S4G). The magnitude of the effect was relatively small, partly because it was a mixed population of transfected and untransfected cells. Live imaging of cells during the wash showed that, in untransfected cells, LDs were independent and highly mobile, whereas in cells overexpressing GRAF1a they remained as clusters and were relatively immobile (Fig. 8G and supplementary material Movie 4).

GRAF1a constructs in which the PH, GAP or SH3 domains were deleted still caused LD clumping after the washout of fatty acids (supplementary material Fig. S4H). However, LDs could spread when a GRAF1a-ΔBAR construct was overexpressed (Fig. 8A,D,E). The capacity of GRAF1a to prevent the redistribution of LDs during the washout of fatty acids from overloaded cells is thus specific, and relies on the presence of the BAR domain.

During LD growth and during lipolysis, GRAF1a therefore causes the association of LDs and restricts their mobility. This accelerates individual LD growth during fatty acid uptake, and retards lipolysis upon washout.

## DISCUSSION

We have identified GRAF1a as the predominant GRAF1 isoform expressed in neonates, whereas GRAF1b and GRAF1c are enriched in adults. In contrast to GRAF1b and GRAF1c, GRAF1a is seldom found on dynamic tubules, but mostly associates with LDs. This is true for tagged and untagged GRAF1a in a variety of cell lines and primary cells. Endogenous GRAF1a expression is, however, highly restricted, because apart from primary glial cells, none of the cell lines we examined (3T3-L1, HeLa, NIH 3T3, U-87 MG, SH-SY5Y, T84, Co115, BSC1 and RPE cells) expressed GRAF1a (our unpublished data). We were, however, able to show enrichment of endogenous GRAF1a in LD fractions isolated from oleic-acid-fed primary glial cells.

Our results show that it is the GRAF1a-specific hydrophobic linker that mediates its targeting to LDs. Similarly, sequences of 30–40 residues containing hydrophobic amino acids interspersed with helix-breaking residues (serine, proline, cysteine and glycine) were found in other LD-associated proteins. In recent years, their number has increased to include DGAT2 (Kuerschner et al., 2008; McFie et al., 2011), NSDHL (Caldas and Herman, 2003), lipin-1γ (Wang et al., 2011b), 17βHSD11 (Horiguchi et al., 2008), GPAT4 (Wilfling et al., 2013), LPCAT1 and LPCAT2 (Moessinger et al., 2011), caveolins (Fujimoto et al., 2001; Ostermeyer et al., 2004; Pol et al., 2001), Rdh10 and LRAT (Jiang and Napoli, 2012; Jiang and Napoli, 2013), AUP1 (Jo et al., 2013; Spandl et al., 2011), UBXD8 (Zehmer et al., 2009), ALDI (Turró et al., 2006), AAM-B and CYB5R3 (Zehmer et al., 2008). For all of these proteins, this segment has been shown to be required for LD localization, and when it was examined, it has been shown to insert as a hairpin in the hydrophobic lipid layer (Caldas and Herman, 2003; Dupree et al., 1993; Moessinger et al., 2011; Spandl et al., 2011; Stevanovic and Thiele, 2013; Zehmer et al., 2008). It is, however, generally not sufficient, and adjacent residues also contribute to the targeting (Ingelmo-Torres et al., 2009; Jiang and Napoli, 2012; Jiang and Napoli, 2013; Lee et al., 2010; Wang et al., 2011b; Zehmer et al., 2008). In addition, for most of these monotopic LD proteins, an important fraction of the protein resides on alternative compartments, and efficient recruitment to LDs requires lipid feeding of the cells. This is reminiscent of GRAF1a mutants in which the BAR and/or PH domains have been deleted. However, even under resting conditions, full-length GRAF1a shows minimal binding to intracellular membranes other than LDs, suggesting that the BAR and PH domains raise the affinity of GRAF1a for the LD surface. Although protein-based interactions cannot be ruled out (Lemmon, 2004), these domains are mostly known for their capacity to interact with membranes, sometimes showing specificity in terms of lipid composition and/or membrane curvature.

In earlier studies, the BAR-PH domain of GRAF1 has been shown to preferentially bind small liposomes (50-nm diameter) with a preference for phosphatidylinositol-4,5- bisphosphate over other phosphoinositides (Lundmark et al., 2008). It has been proposed that phospholipase D1, a phosphatidylinositol-4,5-bisphosphate-binding protein, is important for LD formation in insulin-stimulated NIH 3T3 cells (Andersson et al., 2006). However, LDs are not known to contain any phosphoinositides (Penno et al., 2013), and given that deletion of the PH domain of GRAF1a only marginally perturbs LD localization, it is unlikely to wholly account for the high-affinity binding to LDs.

Although the lipid composition of the outer monolayer of LDs is reminiscent of the ER in terms of relative ratio of phospholipid species, detailed analysis shows that it is different from any other intracellular membrane (Bartz et al., 2007; Penno et al., 2013). It is enriched in free cholesterol and in phospholipids containing a high proportion of saturated fatty acyl chains (Blouin et al., 2010; McIntosh et al., 2010; Tauchi-Sato et al., 2002). This might be linked to the metabolic status of the LD (Arisawa et al., 2013; Storey et al., 2011), and would allow specific recruitment of different pools of proteins (Ehehalt et al., 2006). In order to understand what determines the high-affinity binding of GRAF1a to LDs, it will be essential to further characterize the lipid composition of LDs in non-adipocyte cells and to examine the cholesterol and fatty acyl chain preferences of GRAF1a.

We have shown that GRAF1a is enriched at junctions between LDs and that its overexpression induces LD aggregation and inhibits lipolysis upon washout of fatty-acid-loaded cells. This results in an increase in basal LD levels and is associated with a decreased LD mobility. These phenotypes are specific to GRAF1a because they are not induced by overexpression of GRAF proteins that do not target LDs. In addition, they have not been reported for the above-mentioned monotopic LD-associated proteins, showing they are not merely the result of the insertion of a hydrophobic hairpin in the outer LD layer.

Although the extent varies with the cell type, clustering of LDs is a common phenotype when cells are incubated with fatty acids (Boström et al., 2005; Garcia et al., 2003; Kuerschner et al., 2008; Murphy et al., 2010; Spandl et al., 2011; Turró et al., 2006; Wang et al., 2011a). In adipocytes, it is regulated by phosphorylation of PLIN1 (Marcinkiewicz et al., 2006; Orlicky et al., 2013). However, most non-adipocyte cells do not express PLIN1. In these, the mechanism leading to LD clumping is not known, although it is prevented if the microtubule network, on which LDs travel, is disrupted (Boström et al., 2005; Orlicky et al., 2013). GRAF1a overexpression reduces LD mobility but also potentiates LD clustering. Indeed, in that case, LDs can also contact each other because of cytoplasmic rearrangements, and, in particular, in the presence of GRAF1a, remain tethered, ultimately leading to extensive LD aggregation. The decreased LD mobility observed upon GRAF1a overexpression could also result from the GRAF1a-induced LD clustering because molecular motors associated with each independent LD within a cluster might counteract each other. However, overexpression of Cav^DGV^, a caveolin 3 mutant that constitutively associates with LDs, inhibits LD mobility and lipolysis but does not induce clustering (Pol et al., 2004). In addition, small mobile LD clusters are observed in PLIN1-transfected HEK293 cells during lipolysis (Orlicky et al., 2013), further suggesting that GRAF1a could independently inhibit LD mobility and induce clustering.

LD clustering would be an efficient means to shield the LD surface from lipases, and allow cells with no role in lipid storage to rapidly retrieve lipids following the dispersion of LDs in the cytosol. In agreement with this, we have shown that overexpression of GRAF1a dramatically inhibits LD spreading when fatty acids are removed from the medium of overloaded cells, and that this goes together with an inhibition of lipolysis.

LD fusion is one of the ways in which LDs could grow. Although fusion of LDs of similar sizes seems to be rare (Ariotti et al., 2012; Boström et al., 2005; Murphy et al., 2010), absorption of small LDs into larger ones might occur more frequently. Overexpression of Fsp27 and Cidea, two adipocyte-specific proteins, leads to LD clustering and promotes the formation of enlarged LDs (Christianson et al., 2010; Liu et al., 2009). Fsp27 and Cidea bridge LDs and allow the transfer of triglycerides from the smaller to the larger LD (Gong et al., 2011). Interestingly, their LD fusogenic activities reside in domains dispensable for LD clustering, suggesting that the two are distinct (Christianson et al., 2010; Jambunathan et al., 2011). We have shown that GRAF1a is enriched at inter-LD contacts and that GRAF1a-induced LD clustering depends on its BAR domain. This is not surprising given that GRAF1a will dimerize through its membrane interaction BAR domain while still presenting hydrophobic insertion sequences on each molecule. However, the BAR-PH domain of GRAF1 can also alter local membrane curvature because it induces liposome tubulation *in vitro* (Lundmark et al., 2008). The presence of a curvature effector on LDs would be consistent with an effect on droplet fusion, as this might well prime an area of the surface, making it fusogenic, as is observed with synaptotagmin during vesicle fusion (Martens et al., 2007). This would be consistent with our observation that GRAF1a also labels intracellular tubules, and that these sometimes extend from the LD surface. Use of lipid monolayers with a composition reflecting the outer LD surface will be needed to examine whether the BAR domain of GRAF1a can alter the biophysical properties of LDs and whether this might contribute to inducing LD clustering/fusion.

In addition to studying GRAF1a overexpression in HeLa cells, we have shown that knocking out GRAF1 decreases the capacity of primary glial cells to accumulate LDs upon incubation with oleic acid. Unlike HeLa cells, LDs of primary glial cells are relatively immobile, and LD clustering does not seem to contribute to the first stages of LD growth. GRAF1a could, however, promote LD accumulation by enhancing fusion between a large LD and smaller ones, which we might not resolve in our microscopy analysis. Alternatively, given that knocking out GRAF1a increases LD mobility, GRAF1a might promote the anchoring of LDs to intracellular structures, which might enhance direct lipid transfer (Fei et al., 2008; Gross et al., 2011; Szymanski et al., 2007).

GRAF1a is enriched in the brains of P7 newborn mice. The LD content of various exocrine glands of rats increases shortly after birth (Nagato, 1993; Nagato and Masuno, 1993). We were, however, unable to correlate GRAF1a expression with an increase in LD levels in the brain. LD accumulation might be very restricted in time and/or space, and future work will hopefully allow understanding of the physiological relevance of GRAF1a expression at this developmental stage. Furthermore, because LDs accumulate in brain tumours and neurodegenerative disorders, it will be interesting to employ mouse models to explore any involvement of GRAF1a under such pathological conditions.

GRAF1 is associated with a variety of diseases. Our results now raise the possibility that it might be linked to the involvement of GRAF1a in the regulation of LD homeostasis. The restricted tissue distribution of GRAF1a probably explains the fact that it has not been identified in previous proteomic screens of LDs. However, interestingly, the BAR-domain-containing proteins amphiphysin 2 and SNX1 have been found in LDs isolated from skeletal muscle (Zhang et al., 2011), and FCHSD2 has been identified in LDs of an insulin-producing β-cell line (Larsson et al., 2012). Further studies will be required to confirm their binding to LDs, examine their contributions to LD properties and understand how a membrane-sculpting and -sensing BAR domain might contribute to LD homeostasis.

## MATERIALS AND METHODS

### Cloning

GRAF1 sequences were amplified from a human brain cDNA library (Clontech Laboratories, Mountain View, CA). PCR fragments were recombined in pDONR201 using the Gateway system (Life Technologies, Carlsbad, CA). GRAF1a linker-SH3 [amino acids 699–814 of human GRAF1a (hGRAF1a)], GRAF1 SH3 (amino acids 669–759 of hGRAF1b), GRAF1a ΔSH3 (amino acids 1–758 of hGRAF1a), GRAF1a ΔGAP (deletion of amino acids 383–568 of hGRAF1a), GRAF1a ΔPH (deletion of amino acids 265–369 of hGRAF1a), GRAF1a ΔBAR (amino acids 241–814 of hGRAF1a) and GRAF1 BAR-PH (amino acids 1–382 of hGRAF1a) were subcloned from pDONR01 hGRAF1a. hGRAF2 was amplified from IMAGE clone 40027832 and recombined in pDONR201. Two mutations (A390G, A393G) were introduced to abolish unwanted recombinations during the LR Gateway reaction. All constructs were verified by sequencing.

For expression in mammalian cells, the entry clones were recombined in modified pCI (Promega, Madison, WI) vectors designed to express proteins with C-terminal EGFP or N-terminal Myc tags.

### Antibodies and reagents

The following commercial antibodies were used: rabbit polyclonal anti-calnexin, anti-PLIN3/TIP47/M6PRBP1 and anti-GFP antibodies from Abcam (Cambridge, UK), and anti-calreticulin antibody from Merck Millipore (Darmstadt, Germany); mouse monoclonal anti-β-tubulin (clone 2-28-33) and anti-c-myc (clone 9E10) antibodies from Sigma (St Louis, MO), anti-Rab8 (clone 4, Rab8), anti-Rab11 (clone 47, Rab11) and anti-GM130 (clone 35, GM130) antibodies from BD Biosciences (Franklin Lakes, NJ), anti-COX IV (clone 1D6E1A8) and anti-transferrin receptor (clone H68.4) antibodies from Life Technologies, anti-β-actin antibody (clone AC-15, Abcam), and anti-PLIN2/ADRP antibody (clone AP 125, PROGEN Biotechnik, Heidelberg, Germany).

The secondary reagents for western blotting were horseradish peroxidase (HRP)-conjugated goat anti-mouse-IgG and anti-rabbit-IgG antibodies (Bio-Rad, Hercules, CA) for analysis of extracts, and HRP–Protein-A (Life Technologies) for immunoprecipitations. The secondary antibodies for immunofluorescence were Alexa Fluor 488, 546 or 647 conjugates of goat anti-mouse-IgG and anti-rabbit-IgG antibodies (Life Technologies).

The following reagents were used: Nile Red, Nocodazole, DAPI and Oil Red O from Sigma; and BODIPY 493/503, BODIPY 558/568 C_12_ and HCS LipidTOX Red from Life Technologies. Unless otherwise stated, cell culture products were from Life Technologies.

### Generating anti-GRAF1 and anti-GRAF2 antibodies

GRAF1b GAP-SH3 (amino acids 370–759 of hGRAF1b) and GRAF2 SH3 (amino acids 718–786 of hGRAF2) were cloned in a modified Gateway-compatible pGex 4T2 plasmid (GE Healthcare Life Sciences, Piscataway, NJ). Proteins were expressed in BL21(DE3) pLysS *E. coli* cells (16 h, 18°C). Cells were lysed in 20 mM HEPES pH 7.4, 500 mM NaCl, 1 mM DTT, Complete cocktail of protease inhibitors (Roche, Penzberg, Germany) by freezing–thawing and sonication. Proteins were purified on glutathione–Sepharose beads, cleaved with thrombin, and further purified by Superdex 75 (GRAF1b GAP-SH3) or Superdex 200 (GRAF2 SH3) gel filtration (GE Healthcare Life Sciences). Proteins were injected into rabbits (Eurogentec, Seraing, Belgium). Final bleed sera were purified by affinity chromatography using HiTrap NHS- activated HP columns (GE Healthcare Life Sciences). Specificity was checked using purified proteins and GRAF1^+/+^ and GRAF1^−/−^ brain extracts.

### Cell lines and transfection

HeLa (ECACC 93021013) and BSC1 (ECACC 85011422) cells were grown in DMEM-GlutaMAX supplemented with 10% foetal bovine serum (FBS, HyClone, Thermo Scientific, Waltham, MA). U-87 MG cells (ECACC 89081402) were grown in a 1∶1 mixture of DMEM and Ham's F-12 with GlutaMAX, 15 mM HEPES and 10% FBS. NIH 3T3 cells (ECACC 93061524) were grown in DMEM-GlutaMAX with 10% newborn calf serum; PC12 cells in RPMI 1640 with 7.5% FBS and 7.5% heat inactivated horse serum; and SH-SY5Y cells in a 1∶1 mixture of MEM and Ham's F-12 with GlutaMAX, 1% non-essential amino acids and 15% FBS. HeLa, U-87 MG and SH-SY5Y cells were transfected using GeneJuice (Merck Millipore) and BSC1 cells using Lipofectamine 2000 (Life Technologies). NIH 3T3 cells and PC12 cells were electroporated using the Neon Transfection system (Life Technologies). Unless otherwise stated, cells were processed 24 h after transfection.

### Primary cultures of glial cells

Cortices of mouse E18 embryos or P0 neonates were dissected in ice-cold EBSS buffer containing 10 mM HEPES and penicillin-streptomycin. Cortices were minced, incubated at 37°C with papain (2 U/ml, Worthington, Lakewood, NJ) and mechanically homogenized. Cells were plated onto poly-D-lysine (50 µg/ml) coated culture dishes or coverslips. Co-cultures of glial cells and neurones were grown in Phenol-Red-free MEM with 20 mM glucose, 25 mM HEPES, 1 mM sodium pyruvate and 10% FBS; glial cells were grown in DMEM-GlutaMAX with 10% heat- inactivated horse serum. Cells were grown for 7 days before fatty acids were added or cells were transfected using Lipofectamine 2000. The transfection was repeated on two consecutive days.

### Incubation of cells with fatty acids

Complexes of oleic acid and BSA were prepared as described previously (Martin and Parton, 2008) and diluted in pre-warmed medium for HeLa cells (1.75 mM), primary glial cells (1.25 mM) or U-87 MG cells (1.25 mM). When required for imaging purposes, BODIPY C_12_ (0.2 µg/ml) or BODIPY 493/503 (0.5 µg/ml) was included in the medium. For feeding of transfected cells, oleic acid was initially added 16 h post-transfection. For chase experiments, the oleic-acid-enriched medium was removed after 24 h, cells were thoroughly washed and then incubated in normal medium for another 24 h. When primary glia were incubated with oleic acid for 48 h, the medium was replaced once at 24 h.

### Western blot and immunoprecipitation

Cells and tissues were lysed in 20 mM HEPES pH 7.4, 150 mM NaCl, 2 mM EDTA, 1% IGEPAL CA-630 (Sigma), supplemented with Complete protease inhibitor cocktail. The protein concentration of the cleared supernatants was determined with a Bradford assay (Bio-Rad). Equal protein amounts were either mixed with sample buffer, heated and loaded on 4–12% NuPAGE Novex Bis-Tris gels (Life Technologies) for western blot analysis, or used for immunoprecipitation. For this, cell (100 µg) or tissue (500 µg) extracts were diluted to 100 µl in lysis buffer, and incubated with 1 µl of purified anti-GRAF1 antibody or of pre-immune serum (3 h, 4°C). Protein G Sepharose 4 Fast Flow beads (GE Healthcare Life Sciences) were added for another 30 min. Beads were washed in lysis buffer before adding sample buffer, heating and western blot analysis of a volume equivalent to 100 µg of starting material.

### Cell fractionation by sucrose density centrifugation

U-87 MG (five 15-cm dishes per condition, two of which had been transfected 24 h before) or primary glial cells (20 10-cm dishes) were incubated with oleic acid for 24 h. Cells were then scraped, washed in PBS and lysed in hypotonic buffer (20 mM Tris-HCl pH 7.4, 1 mM EDTA, with Complete protease inhibitor cocktail) by gentle pipetting using a wide-mouthed tip. After a 10-min incubation on ice, lysates were carefully homogenized using a loose Dounce homogenizer and centrifuged (1000 ***g***, 10 min). The supernatants were collected (input) and diluted to 3 ml and a final concentration of 10% sucrose in hypotonic buffer. These were then overlaid with 10 ml hypotonic buffer and centrifuged in a swing-out rotor (280,000 ***g***, 4 h, no brakes). 1 ml fractions were collected from the top of the gradient. For U-87 MG cells, fractions were directly diluted in sample buffer, heated at 50°C and examined by western blotting. For primary glial cells, 400 µl of each fraction and a corresponding volume of input were precipitated with CHCl_3_-MeOH as follows: 1 vol. of MeOH was added, samples were vortexed, then 1 vol. of CHCl_3_ was added, and samples were again vortexed and incubated at room temperature for 20 min. Samples were then centrifuged (10,000 ***g***, 5 min) and the top aqueous MeOH layer was removed, leaving the interface. 4 vol. of MeOH were then added, and samples were vortexed and centrifuged (13,000 ***g***, 15 min). The supernatants were discarded, and after air-drying, the pellets were resuspended in sample buffer and heated at 50°C before analysis by western blotting.

### Sodium carbonate treatment of membranes

Cells were scraped in medium, spun down and washed in PBS. They were then homogenized in hypotonic buffer (20 mM Tris-HCl pH 7.4, 1 mM EDTA, with Complete protease inhibitor cocktail) using a Dounce homogenizer and incubated on ice for 30 min. Membranes were spun down (100,000 ***g***, 30 min) and resuspended in Na_2_CO_3_ 0.1 M pH 11.5. After a 30 min incubation on ice, samples were centrifuged once again (100,000 g, 30 min), the supernatants were collected, and the membranes were washed once in Na_2_CO_3_. Equivalent volumes of pellets and supernatants were analysed by western blotting.

### GRAF1^−/−^ mouse generation

The *Graf1*/*Arhgap26* gene was disrupted by gene targeting (supplementary material Fig. S3A). A replacement construct was generated using a 2.9-kb BamH1 5′ arm of homology (produced using primers 5′-CTCTGAAGACCTACCTGAGGTAGGGATTTC-3′ and 5′- GACACTCGAGTCTCTTGGTTTTCCAGC-3′) and a 2.7-kb Xba1 3′ arm of homology (produced using primers 5′-TCTAGAGCTGCTCATGAACCACCTGGC-3′ and 5′-TCTAGACACAGGTAGAAAACATC-3′). The targeting vector was linearised and transfected into E14.1 (129Ola) embryonic stem cells by electroporation. Genomic DNA from resultant clones was digested with BamH1 and screened by Southern blotting and hybridisation with a probe generated by PCR using primers 5′-GTGTTCCGGACTCTTGATGGTC-3′ and 5′-CCTACCTCAGGTAGGTCTTCAGAG-3′. Correctly targeted clones were injected into C57BL/6 blastocysts to generate chimaeras, which were then crossed with C57BL/6 mice. Subsequent heterozygotes were interbred to generate GRAF1^−/−^, GRAF1^+/−^ and GRAF1^+/+^ mice. All animal work was conducted under Animals (Scientific Procedures) Act 1986, UK, and subject to local ethical approval by MRC Ethical Review Committee.

### Histology analysis

Tissues dissected from 15- to 19-week-old mice were fixed in 10% neutral buffered formalin (Sigma) and paraffin-embedded. Sections (4 µm) were stained with Haematoxylin and Eosin using a Leica Microsystems (Wetzlar, Germany) autostainer ST5020. Pictures were captured with a Nikon (Tokyo, Japan) camera fitted onto a light microscope and using a 5× objective.

For Oil Red O staining, P7 newborn mouse brains were fixed in 10% neutral buffered formalin (72 h, 4°C), incubated in 20% sucrose in PBS (24 h, 4°C) and mounted in OCT (VWR, Radnor, PA). Cryosections (16 µm) were stained with Oil Red O (0.3%, 10 min), rinsed in water (5 min), counterstained with Gill's Haematoxylin, rinsed in water (5 min) and mounted in glycerol in PVA. Sections were visualized under an Olympus (Shinjuku, Japan) BX41 microscope with a Nikon DS-2MV camera and a 50× objective.

### Lipid analysis by TLC

Cells and tissues were homogenized in 20 mM HEPES pH 7.4, 150 mM NaCl, 2 mM EDTA. Lipids were extracted as described previously (Lucken-Ardjomande et al., 2008). Dried lipid films were dissolved in chloroform and volumes equivalent to 100 µg proteins were spotted on TLC Silica Gel 60 plates (Merck Millipore). For major phospholipids, plates were first run in chloroform:ethanol:water:triethylamine (35:40:9:35) until reaching two-thirds of the distance, and were then air-dried; the separation was completed in isohexane:ethylacetate (5:1) (Kuerschner et al., 2005). For cholesterol, plates were run in heptane:ether:acetic acid (18:6:2). Plates were air-dried, bathed in a 3% copper (II) acetate solution in 8% H_3_PO_4_ and heated for 5–15 min at 120°C. Pictures were captured and spot intensities were quantified using ImageJ. Individual spots were identified by comparison with commercial standards.

### Immunofluorescence

Cells grown on coverslips were fixed in 3.2% paraformaldehyde diluted in culture medium (20 min, 37°C). Immunofluorescence was performed using 0.1% saponin for permeabilisation and 5% goat serum for blocking following standard procedures. When needed, Nile Red (2 µg/ml), BODIPY 493/503 (1 µg/ml) or DAPI (1 µg/ml) were included in the secondary antibody dilution. LipidTOX Red staining was performed after the final washes (1∶250, 30 min). Coverslips were mounted in a buffered PVA Glycerol mountant containing 2.5% DABCO (Sigma).

### Super-resolution SIM

SIM was performed on a Zeiss (Goettingen, Germany) ELYRA S.1 using a 63×/1.4 NA oil-immersion objective. The setup consisted of a light-proof incubator, backport ELYRA module, laser rack and PCO (Kelheim, Germany) Edge 4.2 sCMOS camera. Four laser lines were available: 405, 488, 561, and 640 nm. In SIM, a known pattern of low spatial frequency is projected onto the image plane, which, during the recording, is phase-shifted and rotated. The pattern in case of the ELYRA is a grating with constant spatial frequency that varies between the different excitation wavelengths and objectives. We recorded five individual phases and five individual rotations per single plane. Recorded images were then processed with a high-end algorithm to obtain super-resolution SIM images. As indicated in the figure legends, images are either of single focal planes or are presented as maximum intensity projections.

For line-profile analysis of GRAF1a and PLIN3 distribution on the surface of LD clusters, pairs of LDs of similar sizes were selected and visualized on a single focal plane going through the middle of the LD pair. A line dissecting each pair was then drawn and the fluorescence profiles in the red, green and far-red channels were quantified using ImageJ. The distance was normalized to the total width of the pair, and the fluorescence intensities were expressed as a ratio to the maximal value measured. Line profile intensities were averaged for ten pairs of LDs selected from two cells.

### Confocal microscopy

For live imaging, cells were grown on glass-bottomed culture dishes (MatTek, Ashland, MA). Prior to imaging, medium was replaced with Phenol-Red-free DMEM containing 5% FBS and cells were placed in a temperature controlled chamber on the microscope stage with 5% CO_2_ and 100% humidity.

All imaging data were acquired using a fully motorized inverted microscope (Eclipse TE-2000, Nikon) equipped with a CSU-X1 spinning disk confocal head (UltraVIEW VoX, PerkinElmer, Waltham, MA) using a 60× lens (Plan Apochromat V, 1.4 NA, Nikon) under control of Volocity 6.0 (PerkinElmer). 14-bit digital images were acquired with a cooled EMCCD camera (9100-02, Hamamatsu Photonics, Hamamatsu City, Japan). Four 50 mW solid-state lasers (405, 488, 561 and 647 nm, CVI Melles Griot, Rochester, NY) coupled to individual acoustic-optical tunable filters were used as light source. Rapid one- or two-colour time-lapse images were acquired. As indicated in the figure legends, cells are either shown on a single focal plane or as 2D projections of confocal stacks acquired at 0.25-µm intervals. Representative images of live cells and fixed cells collected in independent experiments are shown.

For each cell, quantitative analysis was performed using the Volocity software, identifying LDs by staining with BODIPY C_12_ or BODIPY 493/503, or as PLIN2- or PLIN3-positive objects. For each independent experiment, the mean LD volume, total LD volume and mean LD number in transfected and untransfected cells were quantified. These were then averaged for all independent experiments. Details on the numbers of cells analysed are mentioned in the figure legends, and generally correspond to quantifying the properties of a few hundred to a few thousand LDs.

To quantify the proportion of GRAF1a-positive LDs also positive for PLIN2 or for PLIN3, the percentage of GRAF1a–GFP objects with a PLIN2 or PLIN3 signal above background was quantified for each transfected cell. This was then averaged within an experiment and repeated independently for statistical analysis. Only cells co-stained with anti-PLIN2 and anti-PLIN3 antibodies were used.

For quantification of LD velocity, cells were imaged on a single focal plane over a period of 15 min and images were captured at 5-s intervals. Individual BODIPY-C_12_-positive LDs were tracked in transfected and untransfected cells. Relatively confluent cultures were used to minimize contributions of cell movements to LD mobility.

### Statistical data analysis

For all quantifications provided, the mean±s.e.m. is shown. Details on cell numbers and numbers of independent experiments are provided in the figure legends. Unless otherwise stated statistical significance was assessed by performing two-tailed paired Student's *t*-tests (*P<0.05; **P<0.01; ***P<0.001).

## Supplementary Material

Supplementary Material
