# Supplementary material for: GRAF1a is a brain-specific protein that promotes lipid droplet clustering and growth, and is enriched at lipid droplet junctions
Source: J Cell Sci. 2014 Nov 1;127(21):4602–19. doi: 10.1242/jcs.147694 (PMC4215711; doi:10.1242/jcs.147694)
Supplement: Supplementary Material [file supp_127_21_4602__index.html]

GRAF1a is a brain-specific protein that promotes lipid droplet clustering and growth, and is enriched at lipid droplet junctions — Supplementary Material 

# GRAF1a is a brain-specific protein that promotes lipid droplet clustering and growth, and is enriched at lipid droplet junctions

## JCS147694 Supplementary Material

**Files in this Data Supplement:**

- **Supplementary Material**
