# Supplementary material for: GRAF1a is a brain-specific protein that promotes lipid droplet clustering and growth, and is enriched at lipid droplet junctions
Source: J Cell Sci. 2014 Nov 1;127(21):4602–19. doi: 10.1242/jcs.147694 (PMC4215711; doi:10.1242/jcs.147694)
Supplement: Supplementary Material [file supp_127.21.4602_JCS147694.pdf]

Supplementary material

Supplementary material Fig. S1. (A,B) Adult and P7 mouse brain (A) or adult rat brain (B) extracts were immunoprecipitated with an anti-GRAF1 antibody or a pre-immune serum as a control. Samples were analyzed by western blot using an anti-GRAF1 antibody. Bands corresponding to isoforms a, b and c of GRAF1 are indicated by arrows. (A) The anti-GRAF1 immunoprecipitations were performed in duplicate. (C) Images of single focal planes of live PC12, SH-SY5Y, NIH 3T3 and BSC1 cells expressing GRAF1a-GFP, showing it on the surface of large intracellular vesicles. (D-F) Confocal stack projections of U-87 MG (D) and primary glial (E,F) cells transfected with a plasmid encoding untagged GRAF1a and analyzed by immunofluorescence with an anti-GRAF1 antibody and BODIPY 493/503 to visualize LDs. (F) Cells were pre-incubated with oleic acid for 48 h prior to fixation. (G) Western blot analysis of the sensitivity to sodium carbonate extraction of membrane-bound GFP-tagged GRAF1 proteins. Equivalent volumes of pellets (P) and supernatants (S) were loaded. An antibody directed against the Transferrin Receptor (Tfn R), a transmembrane protein, was used as control. * Tfn R multimers. Unlike the BAR and PH domains, the GRAF1a-specific hydrophobic linker confers resistance to sodium carbonate extraction. (H,I) Single focal plane images of HeLa cells transfected with GFP-tagged GRAF1a-ΔPH (H) or GRAF1a-ΔBAR (I) and analyzed by immunofluorescence using antibodies targeting mitochondria (COX IV), the ER (calreticulin) or the Golgi apparatus (GM130). (J,K) HeLa cells, transfected with GFP-tagged GRAF1a-ΔPH (J) or GRAF1a-ΔBAR (K) were incubated with oleic acid for 24 h prior to fixation. Confocal stacks were acquired. (J) BODIPY C_{12} was included in the oleic-acid-enriched medium. (K) LDs were stained with LipidTOX Red. Unlike GRAF1a-ΔPH, lipid loading does not drive the complete recruitment of GRAF1a-ΔBAR to LDs. (C-F, H-K) A boxed area is enlarged at the bottom right of each image. Scale bars: 15 µm. (L) Total extracts of HeLa cells transfected with the indicated GFP-tagged constructs were analyzed by western blot using an anti-GFP antibody. The GRAF1a-ΔSH3-GFP and GRAF1a-ΔGAP-GFP lanes are from a different blot. Cleavage of the GFP is low for all constructs. There is some cleavage at the N-terminus of the GRAF1a-linker-SH3-GFP protein, but this would release GRAF1-SH3-GFP, which is diffuse (Fig. 2C) and would thus only be expected to

contribute to a background cytosolic staining. (M) U-87 MG cells, transfected as indicated with GRAF1a-GFP, GRAF1a-linker-SH3-GFP, GRAF1a-ΔPH-GFP or GRAF1a-ΔBAR-GFP were incubated with oleic acid for 24 h. Cytoplasmic extracts were fractionated by gradient density centrifugation (fractions 1-13) and analyzed by western blot. LDs, detected using an anti-PLIN3 antibody, are enriched in fraction 1 and contain the GFP-tagged GRAF1a proteins. Rab11 was used as a negative control. Transmembrane proteins like the ER protein calnexin do not enter the gradient but are lost in the pellet. M: marker lane.

Supplementary material Fig. S2. (A-C) HeLa cells were transfected with GFP-tagged GRAF1a and analyzed by immunofluorescence using (A) anti-PLIN2 and anti-PLIN3 antibodies, (B) Nile Red, or (C) an anti-PLIN2 antibody. (A) The fraction of GRAF1a-positive droplets also positive for PLIN2 or for PLIN3 was quantified (74 cells analyzed in 5 independent experiments). (B,C) Projections of confocal stacks are shown. A boxed area containing a GRAF1a tubule is enlarged at the bottom right of each image, showing that it remains unstained by the lipid dye and by anti-PLIN2. Scale bars: 5 µm. (D-F) HeLa cells were transfected with Myc-GRAF1a and fixed 48 h later. Cells were then stained with LipidTOX Red, BODIPY 493/503 or with anti-PLIN3 antibodies. The total LD volume (D), the mean LD volume (E) and the total LD number (F) per cell are plotted here for transfected (G1a) and untransfected (CTRL) cells. LipidTOX Red: 75 CTRL and 216 G1a in 4 independent experiments; BODIPY 493/503: 89 CTRL and 159 G1a in 3 independent experiments; PLIN3: 116 CTRL and 235 G1a in 4 independent experiments. (G) HeLa cells were transfected with a plasmid encoding GRAF1a-GFP and fixed 48 h later. The mean PLIN2 and PLIN3 fluorescence intensity ratios between droplets in transfected and untransfected (CTRL) cells were quantified. PLIN2: 13 independent experiments using 181 CTRL and 243 GRAF1a cells; PLIN3: 11 independent experiments using 152 CTRL and 204 GRAF1a cells. (H) HeLa cells were transfected with GFP-tagged GRAF1b and processed for immunofluorescence analysis with anti-PLIN2 and anti-PLIN3 antibodies. Projections of confocal stacks are shown. Boxed areas containing LDs and GRAF1b tubules are enlarged at the bottom right of each image. GRAF1b is not found on LDs. Scale bars: 15 µm. (I-K) HeLa cells transfected with GRAF1a-GFP were incubated with oleic acid and BODIPY C_{12} to follow LD formation. (I) Time-

lapse movies of single focal planes were acquired, starting at the time of oleic acid addition. Still images at early and late time points from areas of two pairs of untransfected (top) and transfected (bottom) cells acquired in two different experiments, showing the reduction in density of BODIPY C_{12} puncta in transfected cells. Scale bars: 5 μm. (J,K) After 1 h, cells were fixed and processed for immunofluorescence analysis with anti-PLIN3 antibodies, showing GRAF1a-induced LD association. (J) Images are projections of confocal stacks. Scale bars: 15 μm. (K) Ratio of the fluorescence intensity of PLIN3 on BODIPY C_{12}-positive LDs between GRAF1a-transfected and untransfected (CTRL) cells, measured in three independent experiments for a total of 106 GRAF1a and 85 CTRL cells. (L,M) HeLa cells, transfected with GFP-tagged GRAF1a were pre-incubated with oleic acid and BODIPY C_{12} for the indicated times. Live cells were examined under a confocal microscope. Projections of confocal stacks are shown. Scale bars: 15 μm. (L) Time lapse images of the boxed areas captured at 20 s intervals in the red channel show the difference in LD mobility between GRAF1a-transfected (Box 2) and untransfected (Box 1) cells. (M) LDs in oleic-acid-fed GRAF1a-transfected cells (surrounded by dashed lines) are more clumped together than in untransfected cells. (N) GFP-transfected HeLa cells were incubated with oleic acid and BODIPY C_{12} for 24 h, fixed and examined by immunofluorescence with anti-PLIN3 antibodies showing LD clustering. (O) HeLa cells transfected with GRAF1a-GFP were first incubated with oleic acid for 24 h and then incubated for 1 h with or without Nocodazole (0.25 μg/ml). Cells were fixed and processed for immunofluorescence analysis, showing that disruption of the microtubule network (β-tubulin) is not sufficient to reverse LD clumping. (N,O) Projections of confocal stacks. Scale bars: 15 μm.

Supplementary material Fig. S3. (A) Schematic diagram showing disruption of the *Graf1/Arhgap26* gene by gene targeting. The structure of part of the GRAF1 locus, the targeting vector and the predicted homologous recombination event are shown. Targeted disruption results in the deletion of a portion of exon 17 and 20 amino acids (VSEIHSLVHRLPEKNRQMLQ) from the GAP domain, with replacement by the neomycin gene. neo, neomycin resistance cassette; B, BamH1; X, Xba1; TK, thymidine kinase cassette. (B) Average weights of GRAF1$^{+/+}$, GRAF1$^{+/-}$ and GRAF1$^{-/-}$ female and male adult mice (n = 4-20 animals per genotype). (C) Haematoxylin and

eosin staining of sections of brain (cortex, hippocampus, cerebellum), lung, liver, kidney, pancreas, spleen and thymus from GRAF1$^{+/+}$ and GRAF1$^{-/-}$ mice. There is no significant difference in tissue architecture between wild-type and knockout animals. Pictures are representative of more than three pairs of littermates. Scale bars: 0.2 mm. (D,E) TLC analysis of lipids isolated from brain, heart, lung and spleen of GRAF1$^{+/+}$ and GRAF1$^{-/-}$ mice (D), or from the brains of three wild-type adult and three wild-type P7 pups (E) for phospholipids (top) or for cholesterol (bottom). (F) Quantification of the lipid spot intensities of (E). (G) Western blot analysis of GRAF2 expression in brain extracts of GRAF1$^{+/+}$ and GRAF1$^{-/-}$ adult mice. Equal loading was confirmed on the same blot using an anti-actin antibody. The result is representative of four independent pairs of animals. (H) HeLa cells were transfected with GRAF2-GFP, fixed after 48 h and analyzed by immunofluorescence with anti-PLIN2 and anti-PLIN3 antibodies. GRAF2 is not found on LDs. (I) Primary glia isolated from GRAF1$^{+/+}$ and GRAF1$^{-/-}$ embryos were fixed and stained with BODIPY 493/503 and DAPI, showing few LDs in cells under resting conditions. (J-K) GRAF1$^{-/-}$ (J) or GRAF1$^{+/+}$ (K) primary glial cells were transfected with untagged GRAF1a, incubated with oleic acid for 48 h, and fixed. Transfected cells were identified by immunofluorescence analysis with an anti-GRAF1 antibody and LDs were visualized with BODIPY 493/503. Transfected cells are surrounded by dashed lines. (H-K) Projections of confocal stacks. Scale bars: 15 μm.

Supplementary material Fig. S4. (A-G) HeLa cells, transfected with untagged GRAF1a (A), GFP (B, G) or GRAF1a-GFP (C-G), were incubated with oleic acid and BODIPY C_{12} for 24 h. Cells were then either directly fixed (Uptake), or fatty acids were first washed out for another 24 h before fixing the cells (Chase). (A) GRAF1a-induced LD clumping after the chase was visualized by immunofluorescence with an anti-GRAF1 antibody and staining LDs with BODIPY 493/503. (B) After the chase, GFP-transfected cells were immunostained with anti-PLIN3 antibodies, showing that transfection with GFP does not cause LD clumping. (C-E) LDs were identified by immunofluorescence with an anti-PLIN3 antibody, and the mean number (C), the mean volume (D) and the total volume (E) of LD clusters per cell were quantified in 7 (Uptake) and 5 (Chase) independent experiments. Cell numbers: Uptake: 110 CTRL, 146 G1a; Chase: 179 CTRL, 141 G1a. (F) Fluorescence intensity ratio of PLIN3 on

BODIPY C_{12}–positive LD clusters between GRAF1a-transfected and control cells, after the uptake and after the chase, quantified from 5 and 4 independent experiments, respectively. Uptake: 88 CTRL, 119 G1a cells; Chase: 166 CTRL, 124 G1a cells. (G) The amounts of triglyerides (TGs) in HeLa cells transfected with GFP (CTRL) or with GRAF1a-GFP (G1a), left untreated (-), after the uptake or after the chase, were analyzed by TLC. The TG spot intensities were measured and normalized by the value associated with the control cells after the uptake (n = 3). Equivalent expression level of GFP and GRAF1a-GFP was checked by western blot analysis of an equal volume of extracts. β-tubulin was used as a loading control on the same blot. (H) HeLa cells were transfected with the indicated GFP-tagged GRAF1a constructs, incubated with oleic acid and BODIPY C_{12} for 24 h, and chased for another 24 h. Cells were fixed and analyzed by immunofluorescence with anti-PLIN3 antibodies showing that neither the SH3, the GAP or the PH domains of GRAF1a are required for promoting LD clustering. (A,B,H) Images are projections of confocal stacks. Transfected cells are surrounded by dashed lines. Scale bars: 15 μm.

Figure S1

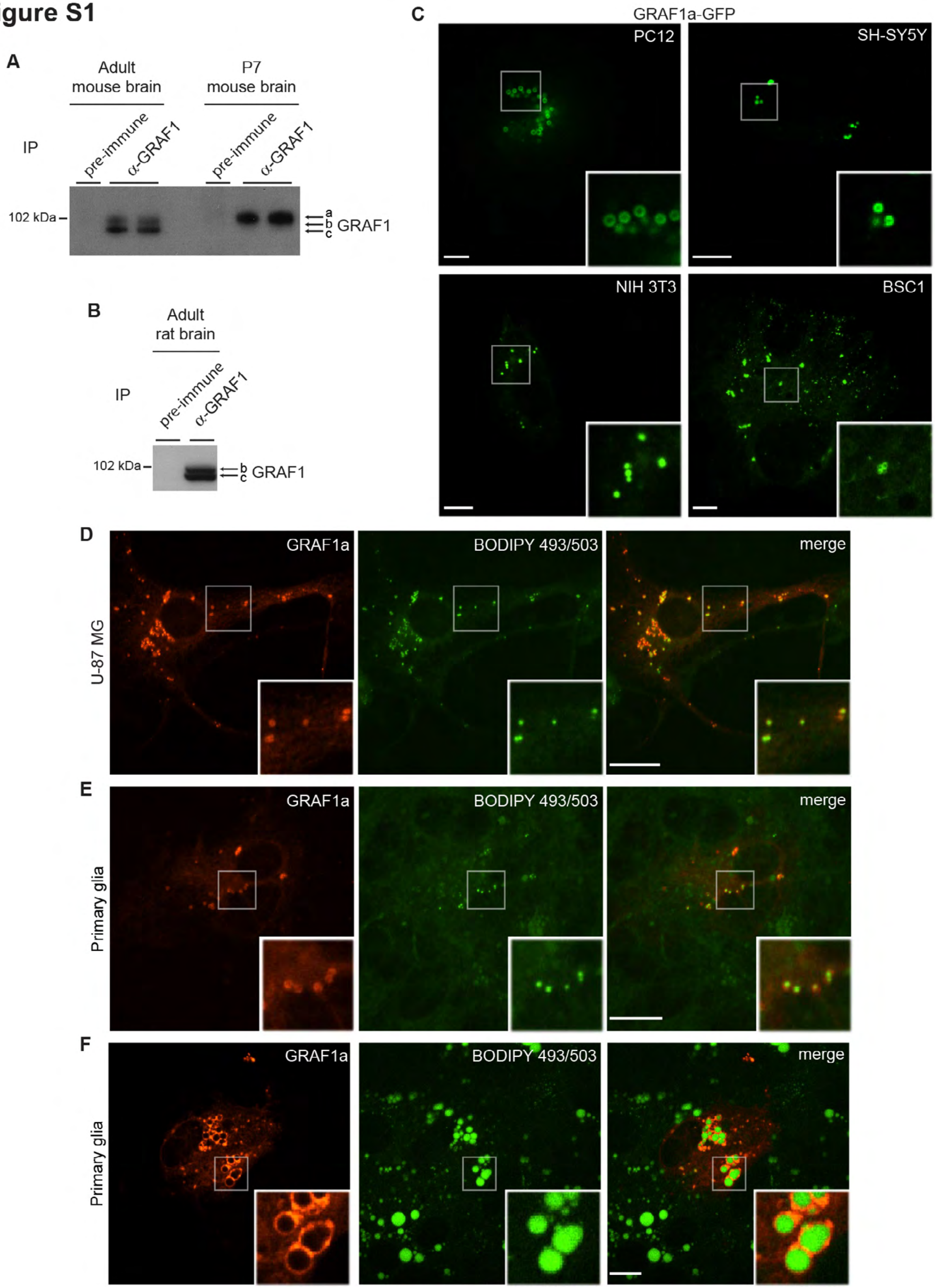
A
Adult mouse brain
P7 mouse brain
IP
pre-immune
α-GRAF1
pre-immune
α-GRAF1
102 kDa
a
b
c
GRAF1
B
Adult rat brain
IP
pre-immune
α-GRAF1
102 kDa
b
c
GRAF1
C
GRAF1a-GFP
PC12
SH-SY5Y
NIH 3T3
BSC1
D
U-87 MG
GRAF1a
BODIPY 493/503
merge
E
Primary glia
GRAF1a
BODIPY 493/503
merge
F
Primary glia
GRAF1a
BODIPY 493/503
merge

(Figure S1)

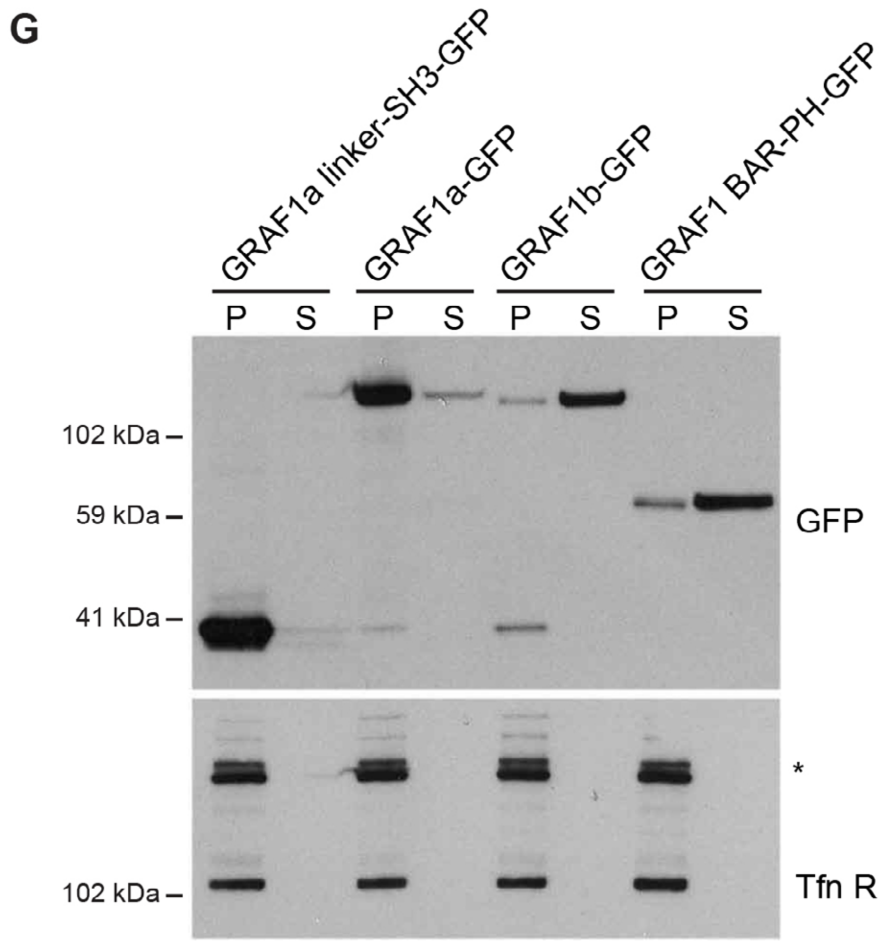
G
GRAF1a linker-SH3-GFP
GRAF1a-GFP
GRAF1b-GFP
GRAF1 BAR-PH-GFP
P
S
P
S
P
S
P
S
102 kDa
59 kDa
41 kDa
GFP
*
102 kDa
Tfn R

(Figure S1)

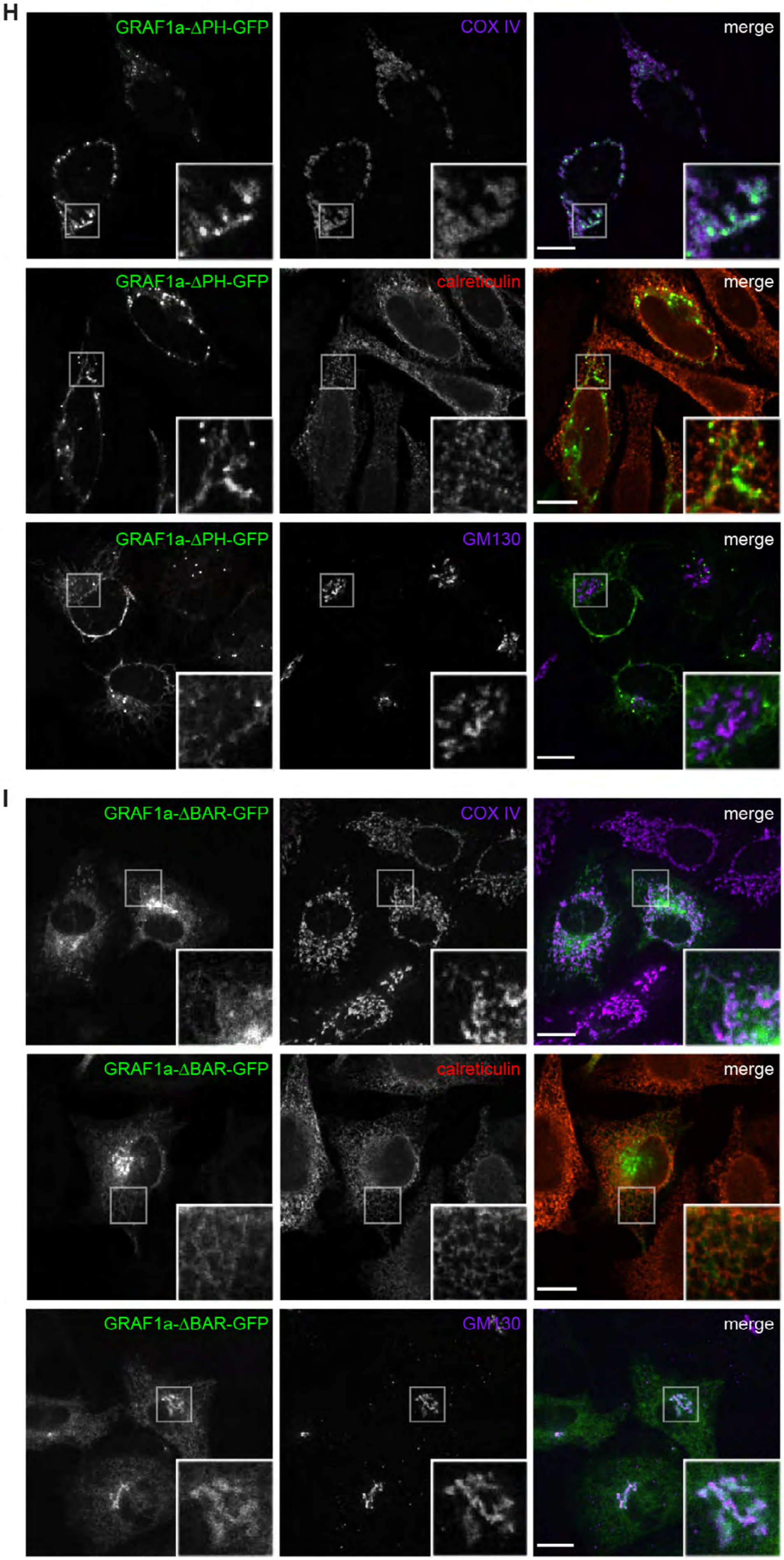
H
GRAF1a-ΔPH-GFP
COX IV
merge
GRAF1a-ΔPH-GFP
calreticulin
merge
GRAF1a-ΔPH-GFP
GM130
merge
I
GRAF1a-ΔBAR-GFP
COX IV
merge
GRAF1a-ΔBAR-GFP
calreticulin
merge
GRAF1a-ΔBAR-GFP
GM130
merge

(Figure S1)

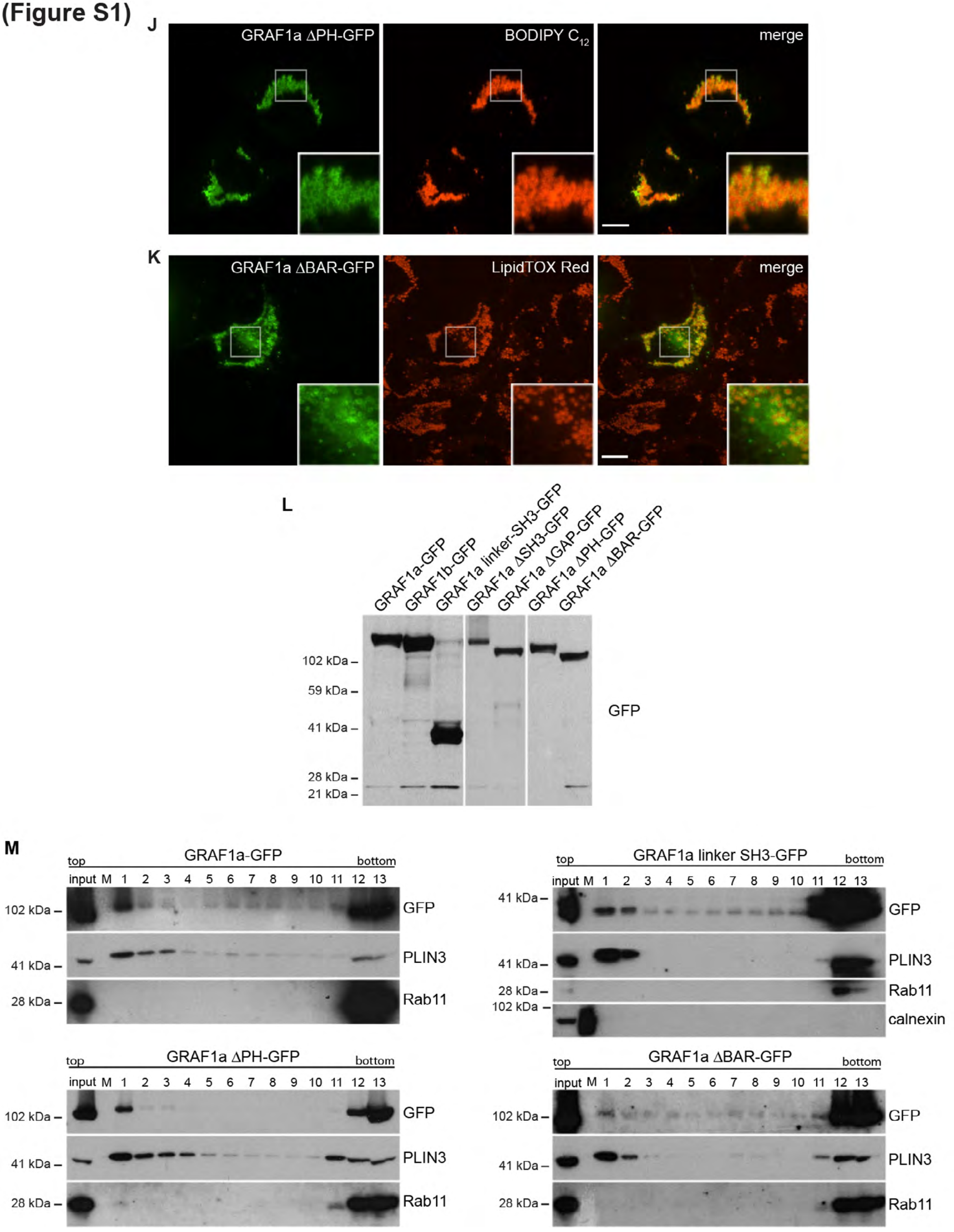
J
GRAF1a ΔPH-GFP
BODIPY C12
merge
K
GRAF1a ΔBAR-GFP
LipidTOX Red
merge
L
GRAF1a-GFP
GRAF1b-GFP
GRAF1a linker-SH3-GFP
GRAF1a ΔSH3-GFP
GRAF1a ΔGAP-GFP
GRAF1a ΔPH-GFP
GRAF1a ΔBAR-GFP
102 kDa
59 kDa
41 kDa
28 kDa
21 kDa
GFP
M
top
GRAF1a-GFP
bottom
input M 1 2 3 4 5 6 7 8 9 10 11 12 13
102 kDa
41 kDa
28 kDa
GFP
PLIN3
Rab11
top
GRAF1a linker SH3-GFP
bottom
input M 1 2 3 4 5 6 7 8 9 10 11 12 13
41 kDa
41 kDa
28 kDa
102 kDa
GFP
PLIN3
Rab11
calnexin
top
GRAF1a ΔPH-GFP
bottom
input M 1 2 3 4 5 6 7 8 9 10 11 12 13
102 kDa
41 kDa
28 kDa
GFP
PLIN3
Rab11
top
GRAF1a ΔBAR-GFP
bottom
input M 1 2 3 4 5 6 7 8 9 10 11 12 13
102 kDa
41 kDa
28 kDa
GFP
PLIN3
Rab11

Figure S2

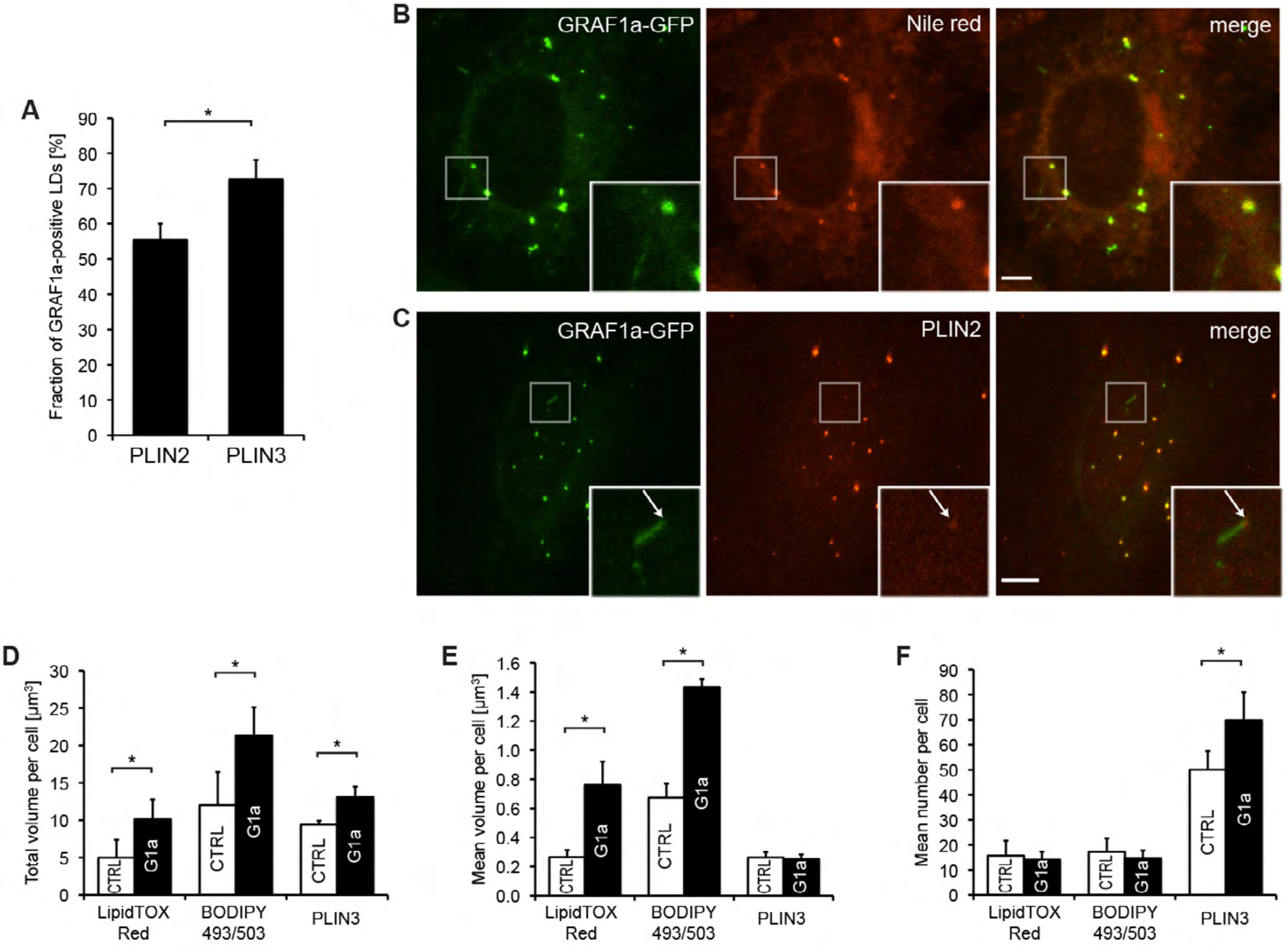

G

	Mean fluorescence intensity ratio GRAF1a / CTRL
PLIN2	1.025 +/- 0.017
PLIN3	0.970 +/- 0.024

H

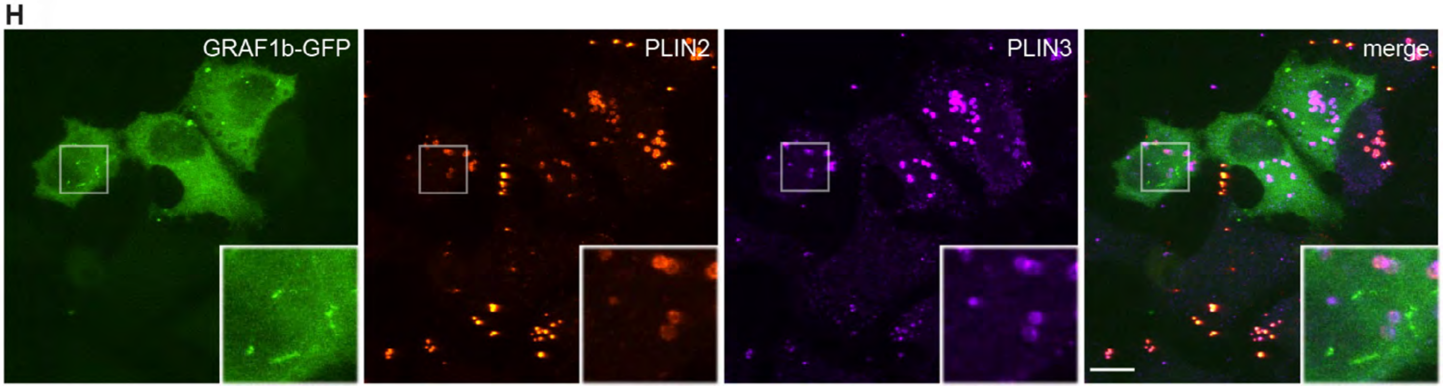

(Figure S2)

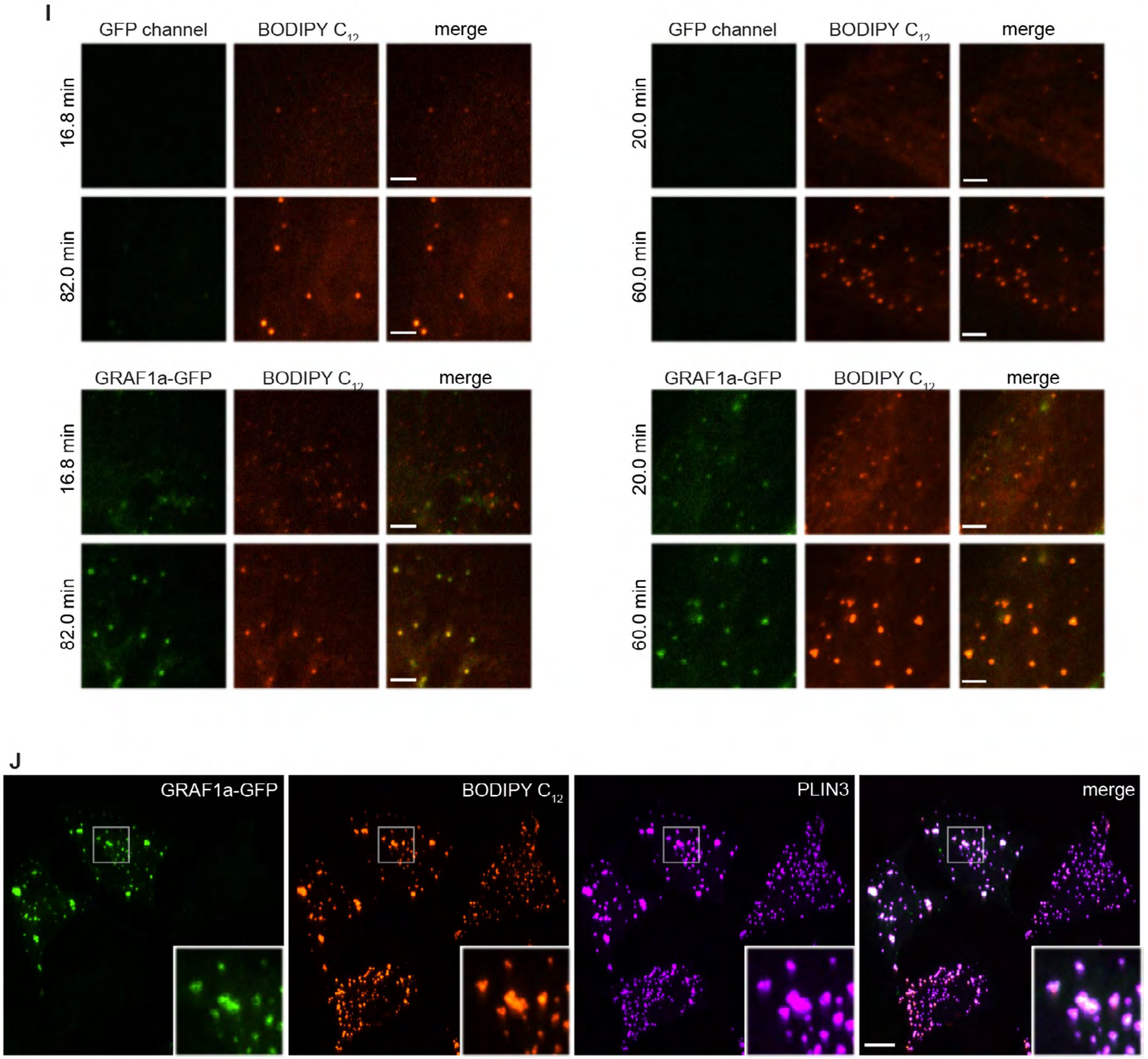

K

	Mean fluorescence intensity ratio GRAF1a / CTRL
PLIN3	1.095 +/- 0.029

(Figure S2)

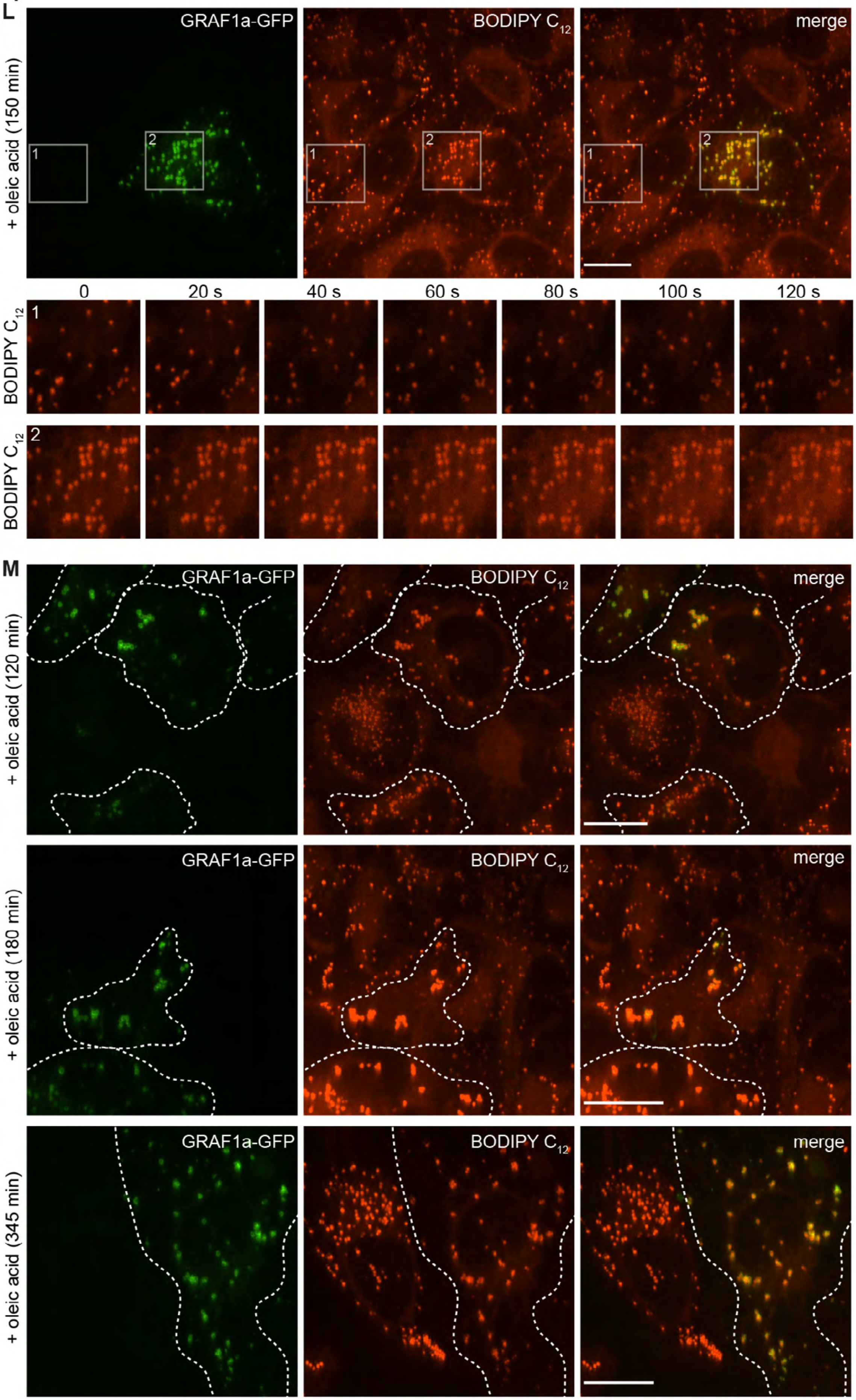
L
GRAF1a-GFP
BODIPY C12
merge
+ oleic acid (150 min)
1
2
0
20 s
40 s
60 s
80 s
100 s
120 s
BODIPY C12
M
+ oleic acid (120 min)
+ oleic acid (180 min)
+ oleic acid (345 min)

(Figure S2)

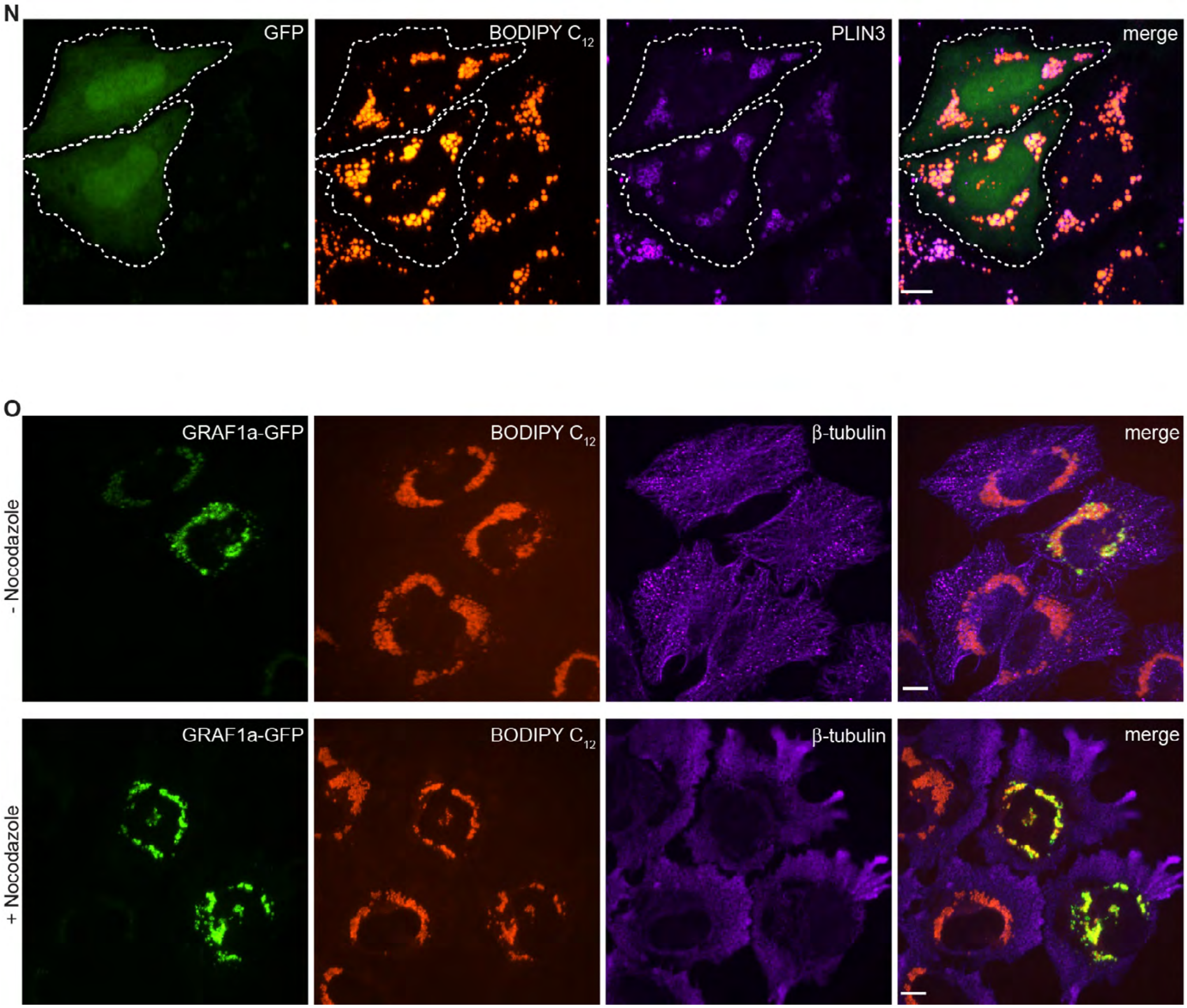
N
GFP
BODIPY C12
PLIN3
merge
O
GRAF1a-GFP
BODIPY C12
β-tubulin
merge
- Nocodazole
+ Nocodazole

Figure S3

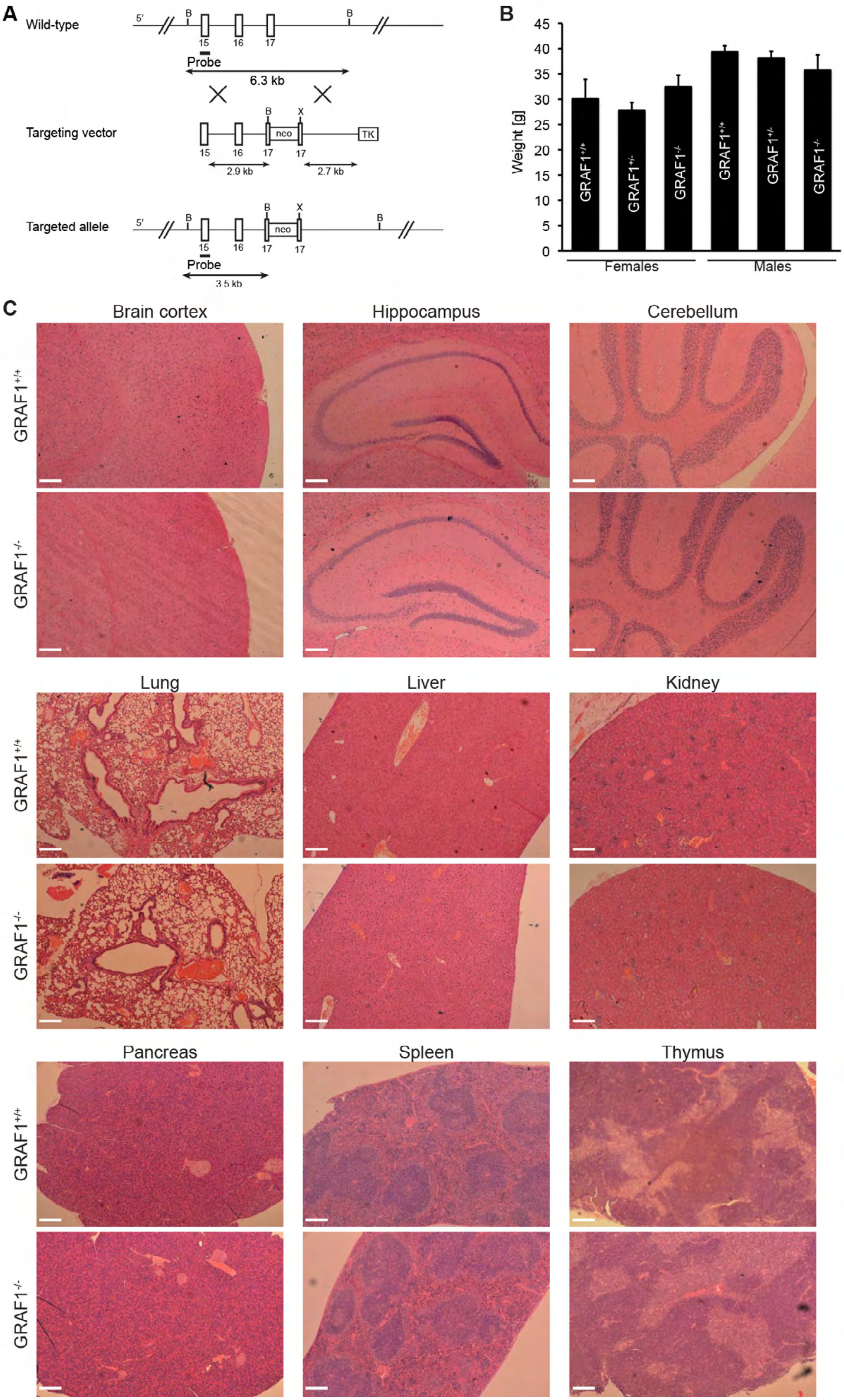
A
Wild-type
5'
B
B
15
16
17
Probe
6.3 kb
Targeting vector
B
X
nco
TK
15
16
17
17
2.9 kb
2.7 kb
Targeted allele
5'
B
B
X
B
nco
15
16
17
17
Probe
3.5 kb
B
Weight [g]
45
40
35
30
25
20
15
10
5
0
GRAF1+/+
GRAF1+/-
GRAF1-/-
GRAF1+/+
GRAF1+/-
GRAF1-/-
Females
Males
C
Brain cortex
Hippocampus
Cerebellum
GRAF1+/+
GRAF1-/-
Lung
Liver
Kidney
GRAF1+/+
GRAF1-/-
Pancreas
Spleen
Thymus
GRAF1+/+
GRAF1-/-

(Figure S3)

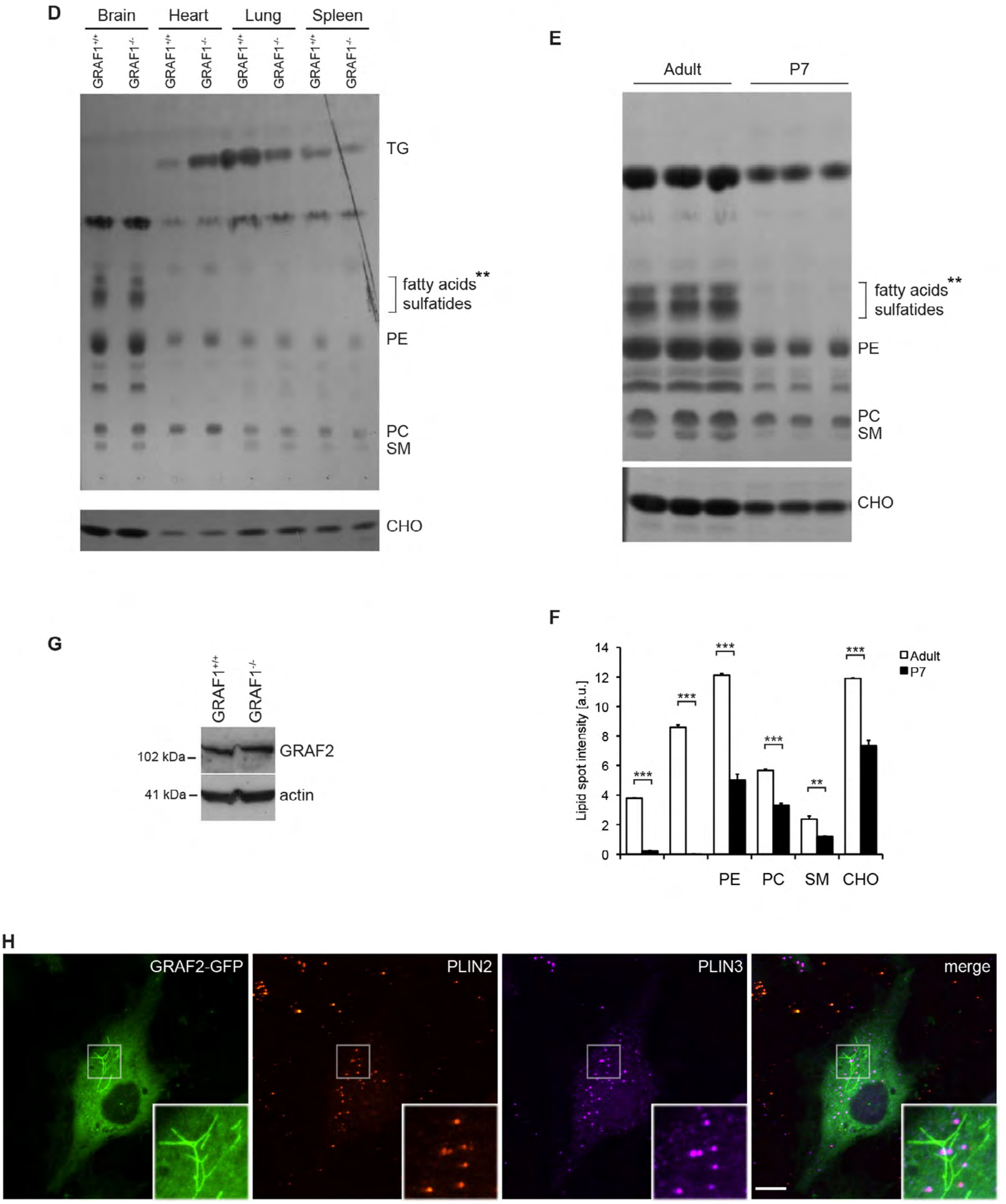
D
Brain
Heart
Lung
Spleen
GRAF1+/+
GRAF1-/-
TG
fatty acids**
sulfatides
PE
PC
SM
CHO
E
Adult
P7
fatty acids**
sulfatides
PE
PC
SM
CHO
G
GRAF1+/+
GRAF1-/-
102 kDa
41 kDa
GRAF2
actin
F
Lipid spot intensity [a.u.]
14
12
10
8
6
4
2
0
Adult
P7
***
**
PE
PC
SM
CHO
H
GRAF2-GFP
PLIN2
PLIN3
merge

(Figure S3)

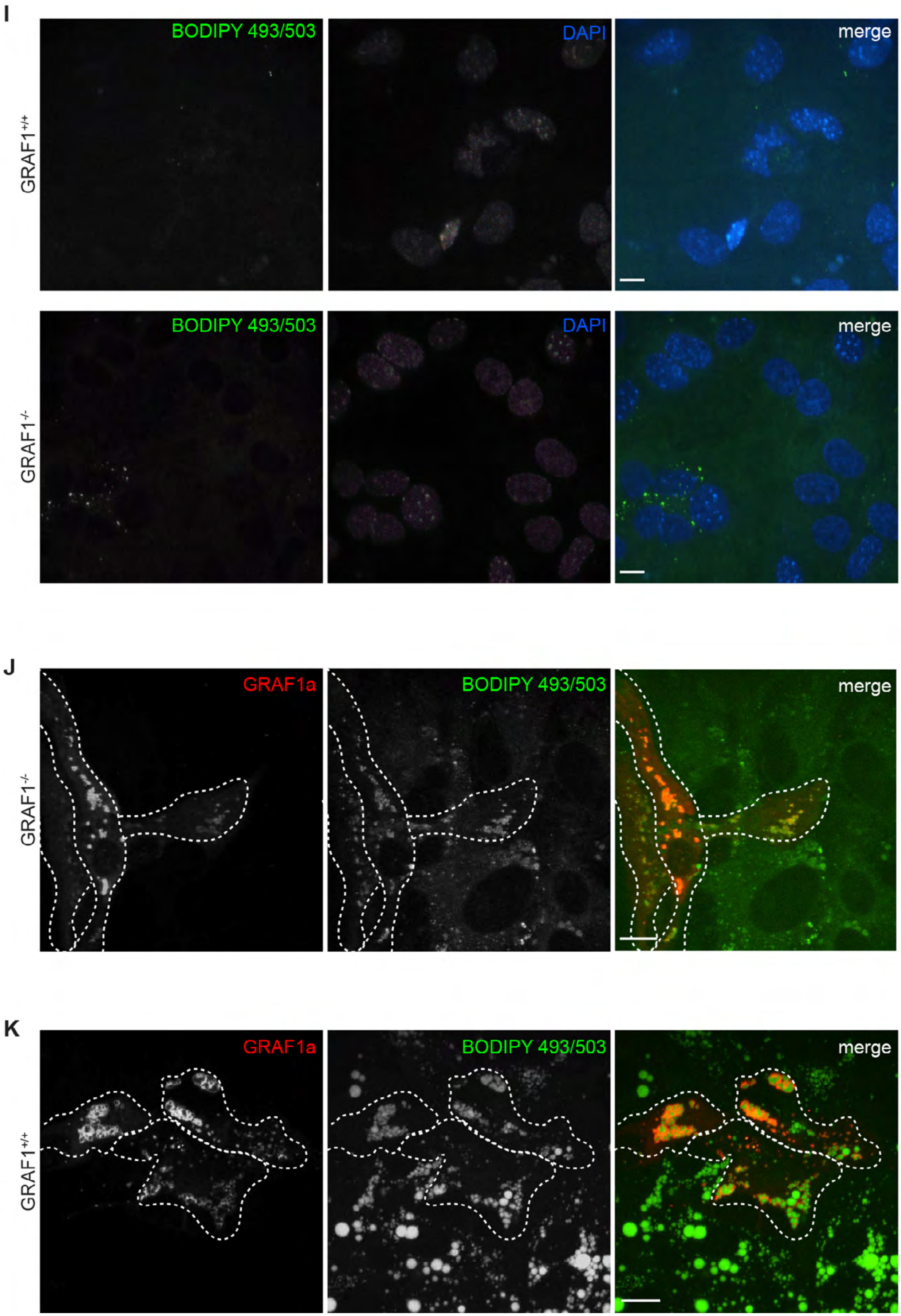
I
BODIPY 493/503
DAPI
merge
GRAF1+/+
BODIPY 493/503
DAPI
merge
GRAF1-/-
J
GRAF1a
BODIPY 493/503
merge
GRAF1-/-
K
GRAF1a
BODIPY 493/503
merge
GRAF1+/+

Figure S4

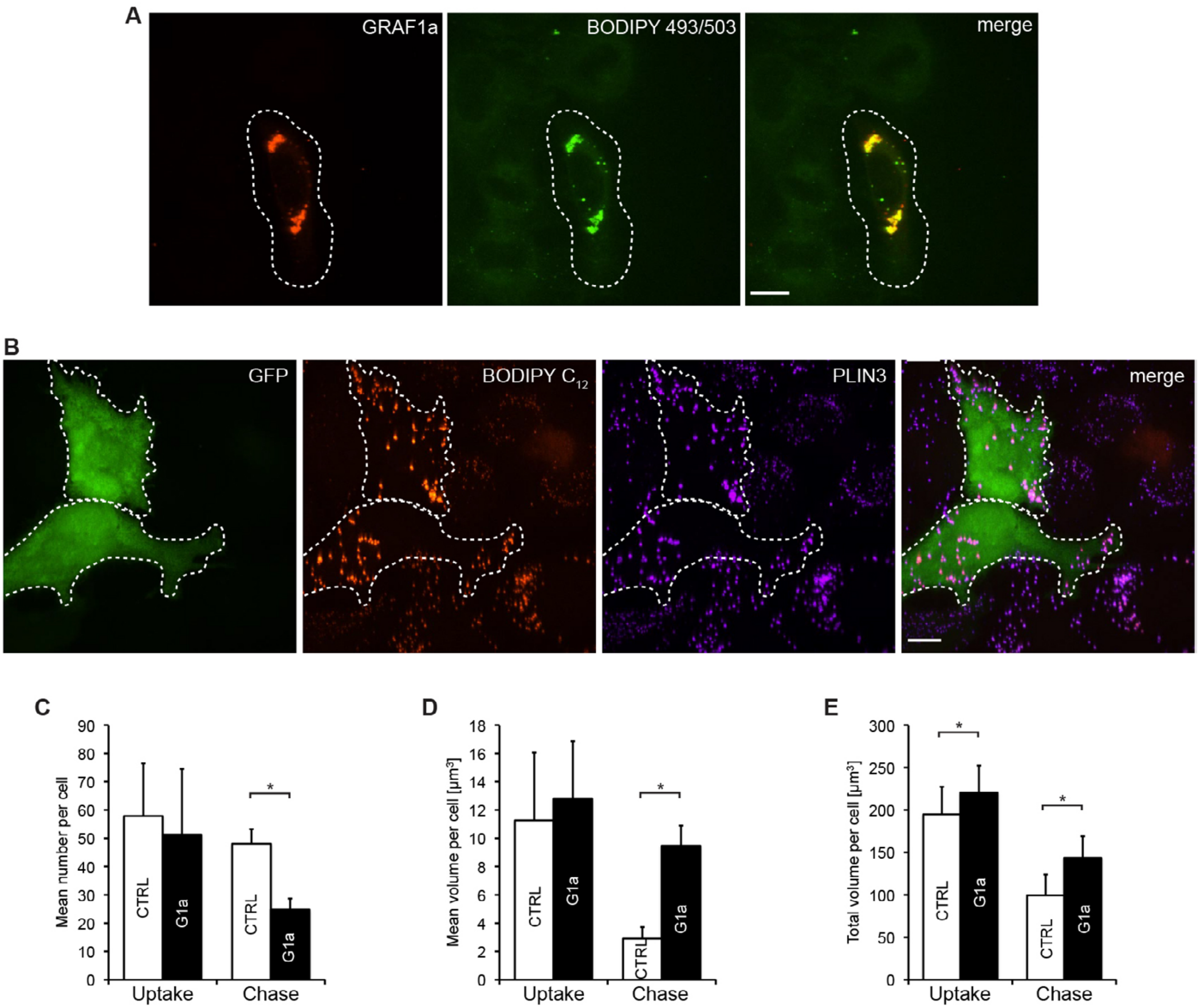

F

	Mean PLIN3 fluorescence intensity ratio GRAF1a/CTRL
Uptake	1.041 +/- 0.039
Chase	1.085 +/- 0.045

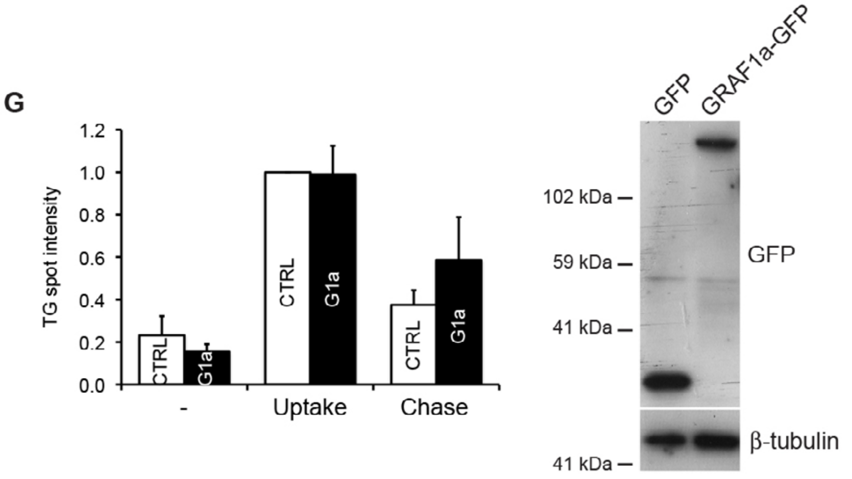

(Figure S4)

H

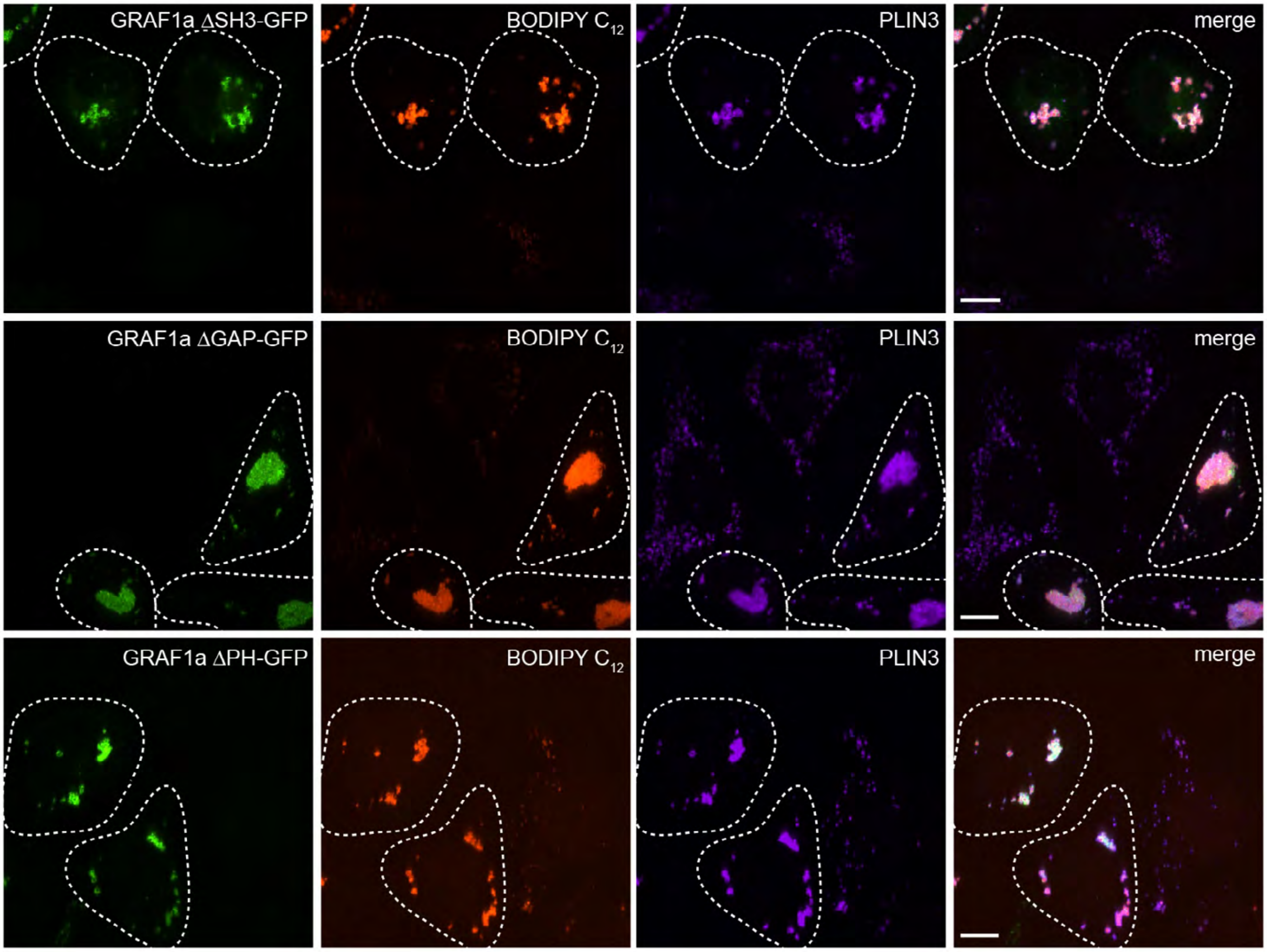
GRAF1a ΔSH3-GFP
BODIPY C_{12}
PLIN3
merge
GRAF1a ΔGAP-GFP
BODIPY C_{12}
PLIN3
merge
GRAF1a ΔPH-GFP
BODIPY C_{12}
PLIN3
merge

Supplementary material movie 1. GRAF1a is found on newly formed LDs. Time-lapse video of HeLa cells transfected with GFP-tagged GRAF1a upon addition of oleic acid and BODIPY C_{12} to follow LD formation. Still images were captured on a single focal plane every 30 s for a total of 1 h and are played here at 12 frames per second. The BODIPY C_{12} fluorescence is shown, and cells transfected with GRAF1a-GFP are delimited by dashed lines. Scale bar: 15 μm.

Supplementary material movie 2. Overexpression of GRAF1a decreases LD mobility. Time-lapse video of HeLa cells, pre-incubated for 5 h with oleic acid and BODIPY C_{12}. Still images were captured on a single focal plane every 10 s for a total of 15 min and are played here at 12 frames per second. The BODIPY C_{12} fluorescence is shown, and cells transfected with GRAF1a-GFP are delimited by dashed lines. Scale bar: 15 μm.

Supplementary material movie 3. LDs in GRAF1$^{-/-}$ primary glia are more mobile than in GRAF1$^{+/+}$ cells. Time-lapse videos of GRAF1$^{+/+}$ (left) and GRAF1$^{-/-}$ (right) cells, pre-incubated with oleic acid for 10 h and stained with BODIPY 493/503. Still images were captured on a single focal plane every 5 s for a total of 5 min and are played here at 6 frames per second. Boxed regions are magnified at the bottom right of each movie. Scale bars: 15 μm.

Supplementary material movie 4. Overexpression of GRAF1a promotes LD clumping and decreases LD mobility during the wash of fatty acids from overloaded cells. Time-lapse video of HeLa cells, pre-incubated with oleic acid and BODIPY C_{12} for 8 h, starting upon fatty acid removal from the medium. Still images were captured on a single focal plane every 10 min for a total of 9.5 h and are played here at 6 frames per second. The BODIPY C_{12} fluorescence is shown, and cells transfected with GRAF1a-GFP are delimited by dashed lines. Scale bar: 15 μm.

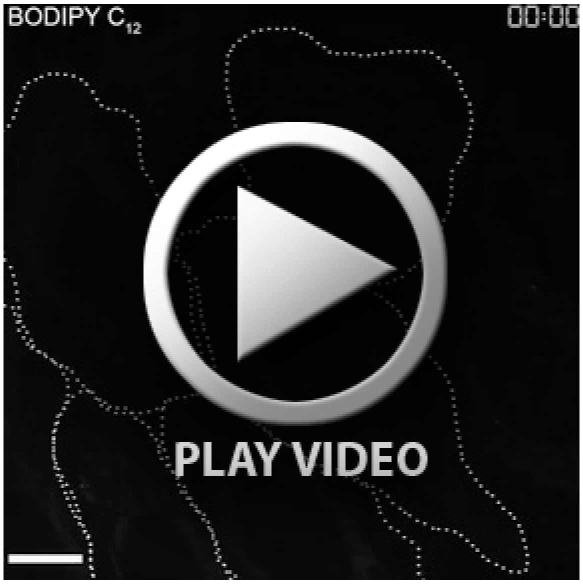

Movie 1.

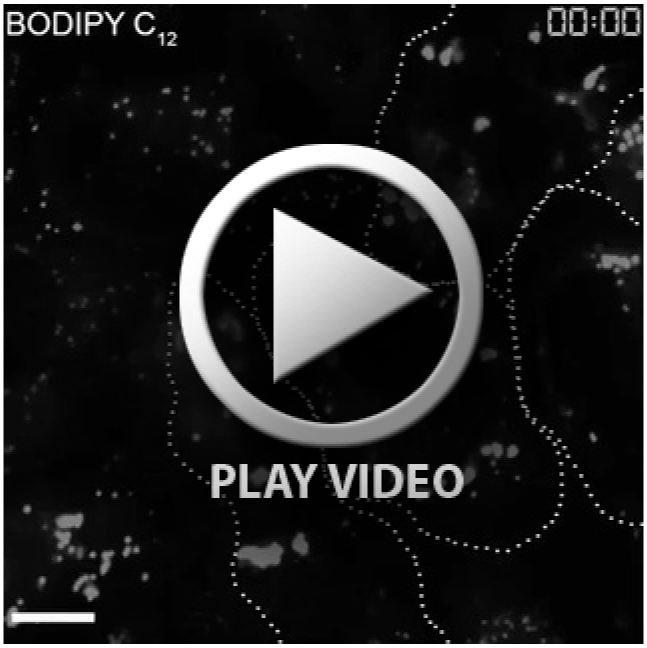

Movie 2.

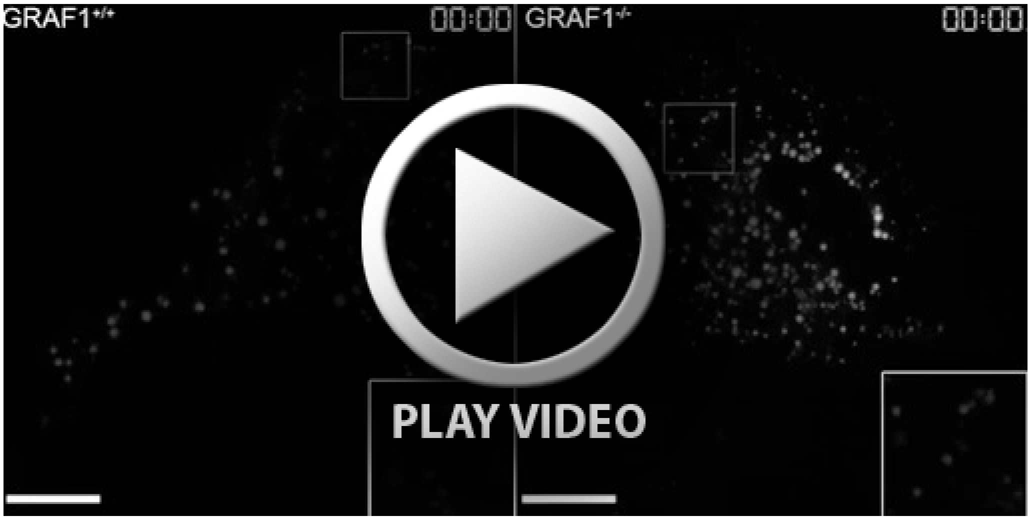

Movie 3.

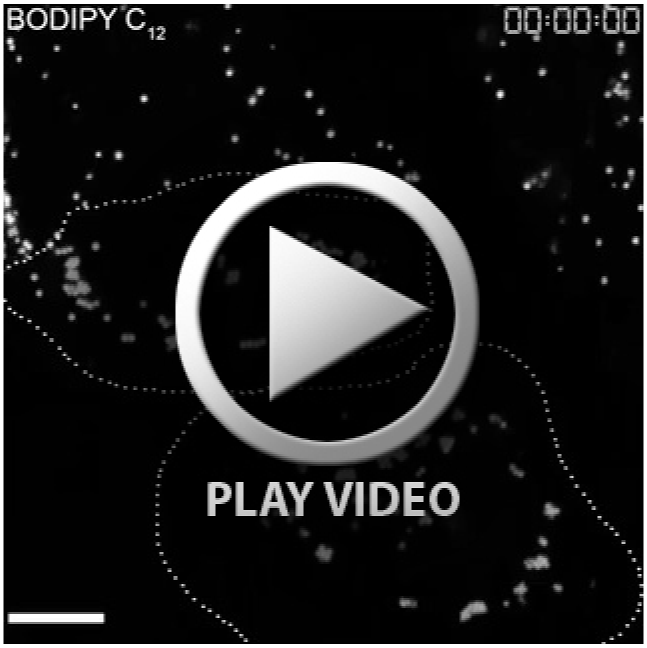

Movie 4.
